# Rapidly Evolving Genes Are Key Players in Host Specialization and Virulence of the Fungal Wheat Pathogen *Zymoseptoria tritici* (*Mycosphaerella graminicola*)

**DOI:** 10.1371/journal.ppat.1005055

**Published:** 2015-07-30

**Authors:** Stephan Poppe, Lena Dorsheimer, Petra Happel, Eva Holtgrewe Stukenbrock

**Affiliations:** Max Planck Research Group Fungal Biodiversity, Max Planck Institute for Terrestrial Microbiology, Marburg, Germany; Wageningen University, NETHERLANDS

## Abstract

The speciation of pathogens can be driven by divergent host specialization. Specialization to a new host is possible via the acquisition of advantageous mutations fixed by positive selection. Comparative genome analyses of closely related species allows for the identification of such key substitutions via inference of genome-wide signatures of positive selection. We previously used a comparative genomics framework to identify genes that have evolved under positive selection during speciation of the prominent wheat pathogen *Zymoseptoria tritici* (synonym *Mycosphaerella graminicola*). In this study, we conducted functional analyses of four genes exhibiting strong signatures of positive selection in *Z*. *tritici*. We deleted the four genes in *Z*. *tritici* and confirm a virulence-related role of three of the four genes *ΔZt80707*, *ΔZt89160* and *ΔZt103264*. The two mutants *ΔZt80707* and *ΔZt103264* show a significant reduction in virulence during infection of wheat; the *ΔZt89160* mutant causes a hypervirulent phenotype in wheat. Mutant phenotypes of *ΔZt80707*, *ΔZt89160* and *ΔZt103264* can be restored by insertion of the wild-type genes. However, the insertion of the *Zt80707* and *Zt89160* orthologs from *Z*. *pseudotritici* and *Z*. *ardabiliae* do not restore wild-type levels of virulence, suggesting that positively selected substitutions in *Z*. *tritici* may relate to divergent host specialization. Interestingly, the gene *Zt80707* encodes also a secretion signal that targets the protein for cell secretion. This secretion signal is however only transcribed in *Z*. *tritici*, suggesting that *Z*. *tritici*-specific substitutions relate to a new function of the protein in the extracellular space of the wheat-*Z*. *tritici* interaction. Together, the results presented here highlight that *Zt80707*, *Zt103264* and *Zt89160* represent key genes involved in virulence and host-specific disease development of *Z*. *tritici*. Our findings illustrate that evolutionary predictions provide a powerful tool for the identification of novel traits crucial for host adaptation and pathogen evolution.

## Author Summary

*Zymoseptoria spp* provides a unique model system to study the underlying genetics of host specialization of plant pathogens. Closely related *Zymoseptoria* species, including the prominent wheat pathogen *Z*. *tritici*, have recently specialized to distinct grass hosts. Positively selected substitutions have played a central role in the acquisition of new host specificities. We have identified a small set of genes showing signatures of positive selection. We demonstrate that three of these four candidate genes play an important role during host infection. Two mutants of *Z*. *tritici* were impaired in virulence; a third mutant showed a hypervirulent phenotype. New protein specificities not only include changes at the amino acid sequence level but also at the level of the protein structure. We conducted a gene replacement experiment to test if mutant phenotypes in *Z*. *tritici* could be complemented by the insertion of orthologous genes from the two closely related species *Z*. *pseudotritici* and *Z*. *ardabiliae*. For two genes, we confirm that the species-specific protein changes are essential for proper protein functioning in *Z*. *tritici*; key traits involved in the evolution of virulence and host specificity of this prominent pathogen can be characterized via a combination of evolutionary predictions and functional analyses.

## Introduction

Host specialization of pathogens can be a strong driver of diversification and speciation [1]. Host-driven speciation implies the specialization of traits involved in host interactions from the initial infection and the defeat of host defenses to within-host nutrient uptake, multiplication and reproduction. Even closely related host species may differ in their repertoire of defense-related genes, as well as their biochemical and physical properties. As a result, distinct selection pressures are imposed on infecting pathogens. Genes affected by divergent selection during host specialization can be recognized in pathogen genomes as outlier loci with an excess of genetic divergence [2]. Positive selection and adaptive changes at the amino acid level are in particular reflected by an accumulation of non-synonymous divergence at the nucleotide level.

In plant pathogens, divergent selection has been documented in several studies showing specialization of genes related to different functions, e.g., genes encoding effector proteins [3], secondary metabolites [4], toxins [5] and genes encoding cell wall-degrading enzymes [6]. The functional implications of divergent selection are, however, poorly understood. Dong and co-workers elegantly demonstrated that one amino acid change in the oomycete effector protein EpiC1 determines target specificity [3]. EpiC1 encodes a protease inhibitor that has evolved under strong positive selection during the divergence of the two plant pathogenic *Phytophthora* species *P*. *infestans* and *P*. *mirabilis* infecting different *Solanum* plants and *Mirabilis jalapa*, respectively. The adaptive changes in EpiC1 directly reflect differences in the protease targets of EpiC1 of *P*. *infestans* and *P*. *mirabilis*, and the study illustrates the direct functional effect of positive selection.

For the fungal wheat pathogen *Zymoseptoria tritici* (*Mycosphaerella graminicola*), the underlying genetics of host-pathogen interaction is poorly understood. Infection of *Zymoseptoria spp*. involves an initial biotrophic phase where infectious hyphae take up readily accessible sugars in the apoplast. Host cell death is induced after approximately two weeks and involves a strong proliferation of hyphae in substomatal cavities and the formation of asexual fruiting bodies, so-called pycnidia [7]. Specialization to wheat likely has involved the acquisition and fixation of adaptive substitutions in key genes playing a role in the molecular interaction between host and pathogen. Several non-characterized genes, including genes encoding both secreted and non-secreted proteins, were found to exhibit signatures of positive selection during divergence from the closest relatives *Z*. *pseudotritici* and *Z*. *ardabiliae* [7–8]. *Z*. *pseudotritici* and *Z*. *ardabiliae* were isolated from wild grasses in the Middle East and are unable to infect the host of *Z*. *tritici* (bread wheat). Divergence of these three species occurred very recently and involved only a few adaptive changes at the genome level [8]. It is likely that these few changes have been instrumental during specialization to distinct hosts. We hypothesize that genes subjected to positive selection during speciation of *Z*. *tritici* likely have played a role in the specialization to wheat. Using genome-wide analyses of non-synonymous to synonymous divergence, we previously identified a set of 27 positively selected genes in *Z*. *tritici* [8]. In this study, we aim to elucidate the underlying role of four selected genes showing increased ratios of non-synonymous to synonymous substitutions. We adopt a reverse genetic approach and show that three of the genes have a strong impact on virulence and reproduction of *Z*. *tritici* during infection of wheat. Besides amino acid changes, we describe structural variation in transcript lengths including the addition of a signal peptide in one *Z*. *tritici*-encoded protein.

## Results

### Selection of candidate genes

We previously conducted a comparative genome study in which we assessed pairwise *K*_*a*_/*K*_*s*_ ratios for more than 9000 aligned genes in *Z*. *tritici*, *Z*. *pseudotritici*, *Z*. *ardabiliae* and *Z*. *passerinii* with the aim of identifying positively selected genes [8]. In the present study, we address the functional relevance of a subset of these positively selected genes in *Z*. *tritici*. We selected the four genes *Zt80707*, *Zt89160*, *Zt103264* and *Zt110804* for which we previously identified signatures of positive selection using two different approaches [8]. *Zt80707* and *Zt103264* were selected from a list of 27 candidates with *K*_*a*_ > *K*_*s*_ (computed according to Nei and Gojobori [9], FDR-adjusted *p*-value < 0.05; Z-test). Both genes show an increased accumulation of non-synonymous substitutions in pairwise comparisons between *Z*. *tritici*-*Z*. *pseudotritici* and *Z*. *tritici*-*Z*. *ardabiliae*. The two genes *Zt89160* and *Zt110804* were selected from the output of a maximum likelihood analysis of gene-wise branch specific *d*_N_/*d*_S_ ratios in the three *Zymoseptoria* species. Both genes show increased *d*_N_/*d*_S_ ratios exclusively in the *Z*. *tritici* branch. These four genes are all located on the core chromosomes in regions with a well-conserved synteny among the three *Zymoseptoria* species (Table 1). None of the genes have previously been characterized functionally in *Z*. *tritici*; however, the gene *Zt89160* is predicted to encode a Regulator of Chromosome Condensation (RCC1) domain [10] and *Zt110804* encodes a hypothetical protein containing a proline-rich region predicted to be involved in binding with other proteins [11]. The genes *Zt80707* and *Zt103264* show no homology to known proteins.

**Table 1:** Candidate genes and genomic coordinates in *Z*. *tritici*.

Gene ID	Location	Length [aa]	*K*_*a*_/*K*_*s*_ *Z*.*tritici* vs. *Z*.*pseudotritici*	Predicted function
80707	Chr_5:0657996–0658613	125	5.825	Unknown / secreted
89160	Chr_1:2089079–2090359	394	5.392	RCC1 domain
103264	Chr_2:1992714–1993521	173	2.303	Unknown
110804	Chr_9:1317146–1318829	317	3.533	Kinase

**Fig 1:**
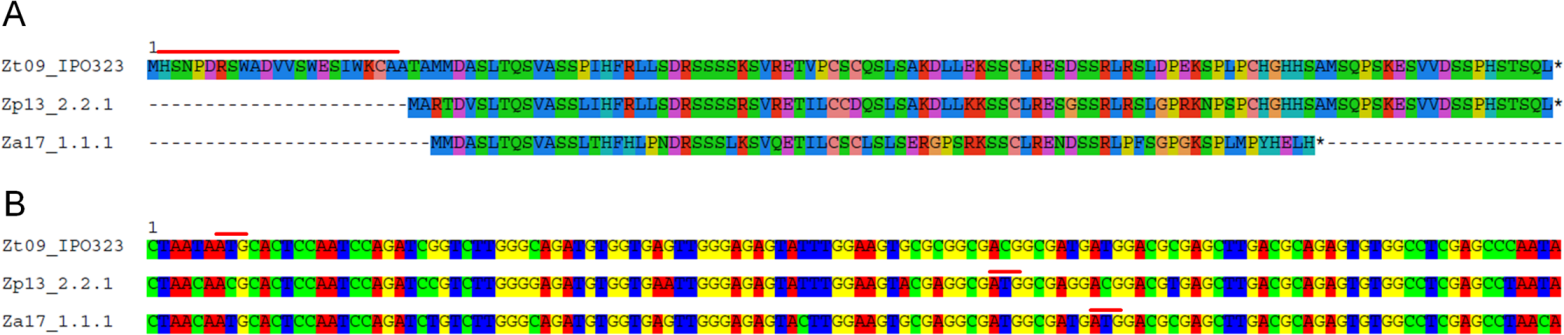
Variation at the nucleotide and protein levels between orthologs of *Zt80707*. A) Alignment of the protein Zt80707 of *Z*. *tritici* (Zt) and orthologs in the sister species *Z*. *pseudotritici* (Zp) and *Z*. *ardabiliae* (Za). The alignment demonstrates the difference in length of the orthologous proteins. The additional 25 amino acids at the N-terminal end of the *Z*. *tritici* ortholog encodes a weak signal peptide (red line). B) Nucleotide alignment of the genomic region encoding the Zt80707 protein including the nucleotide sequence encoding the signal peptide in *Z*. *tritici* and the corresponding up-stream sequences in *Z*. *pseudotritici* and *Z*. *ardabiliae*. Different start codon positions in *Z*. *tritici*, *Z*. *pseudotritici* and *Z*. *ardabiliae* identified by RACE-PCR are indicated with red lines.

Gene alignments showed that the structure of the reading frames of *Zt89160* and *Zt110804* are conserved between the three *Zymoseptoria* species with identical start and stop codon positions. However, we verified the open-reading frames of *Zt80707* and *Zt103264* by Rapid Amplification of cDNA Ends (RACE-PCR) in *Z*. *tritici*, *Z*. *pseudotritici* and *Z*. *ardabiliae* as the orthologous sequences for these two genes had different start and stop codon positions. Based on sequencing of transcripts, we revealed significant differences in transcript lengths for those two genes among the three *Zymoseptoria* species (Figs 1 and S1). According to these structural differences, we asked whether the genes are located next to transposons or in regions with frequent re-arrangements. To this end, we analyzed the four loci in *Z*. *tritici* (isolate IPO323) and compared them with the orthologous loci in the *Z*. *pseudotritici* isolate STIR04_2.2.1 (here termed Zp13) and the *Z*. *ardabiliae* isolate STIR04_1.1.1 (here termed Za17) [8,12]. We found transposable and repetitive elements in the vicinity of the neighboring genes (<30kbp) but none directly associated (<2kbp) with the candidate genes (S2 Fig). We next re-analyzed the extent of positive selection in the gene models of *Zt80707* and *Zt103264* corrected by RACE-PCR in *Zymoseptoria spp* using a maximum likelihood approach. We applied a branch model to obtain maximum likelihood estimates of branch-specific ω values (*d*_N_/*d*_S_ ratios) [13]. This approach allowed us to compare the branch-specific accumulation of non-synonymous to synonymous divergence in the individual *Zymoseptoria* lineages for each gene. An ω value above 1 indicates that a gene has been subjected to positive selection in a particular branch of a phylogenetic tree during the divergence of lineages. Our analyses confirm previous results using the Nej and Gojobori *K*_*a*_/*K*_s_ estimates [8] and show that all four genes have evolved under positive selection during divergence of *Z*. *tritici* (*Zt89160* and *Zt110804*) or during divergence of all three *Zymoseptoria* lineages (*Zt80707* and *Zt103264*) according to branch specific d_N_/d_S_ ratios (S3 Fig).

### *Zt80707* encodes a signal peptide solely translated in *Z*. *tritici*

**Fig 2:**
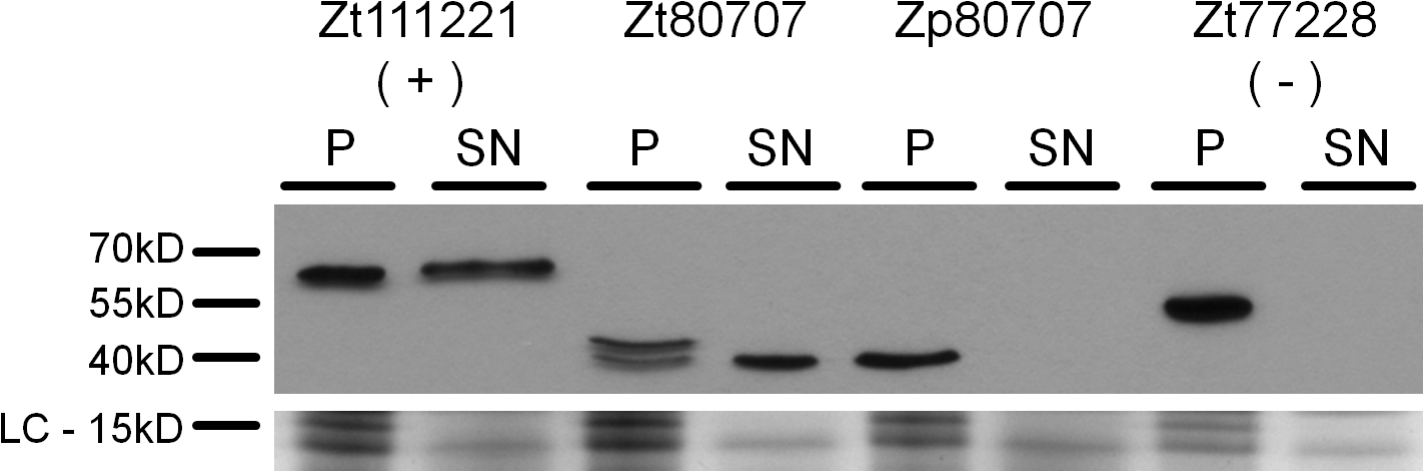
*Zt80707* encodes a signal peptide targeting the protein for cell secretion in *Z*. *tritici*. Western blot analyses of the proteins Zt111221-GFP (secreted LysM effector, positive control (+)), Zt80707-GFP (*Z*. *tritici*), Zp80707-GFP (*Z*. *pseudotritici*) and Zt77228-GFP (non-secreted protein, lysis control (-)) detected in the pellet fraction (P) and the cell culture supernatant (SN) with an anti-GFP antibody. The presence of the GFP tagged gene products in the supernatant demonstrates the secretion of the Zt80707 protein and the functioning of the predicted signal peptide of Zt80707. As Loading Control (LC) a Coomassie stained SDS gel is included to show comparable amounts of protein loaded in the lanes of the different samples.

Remarkably, the gene *Zt80707* showed different transcript lengths among the three species, *Z*. *tritici*, *Z*. *pseudotritici* and *Z*. *ardabiliae*. For the orthologous transcripts *Zp80707* in *Z*. *pseudotritici* and *Za80707* in *Z*. *ardabiliae*, the transcription start sites are 69 and 75 nucleotides downstream of the start site in *Z*. *tritici*, respectively. The differing transcripts lengths between *Zymoseptoria* species were confirmed using RT-PCR analysis (S4 Fig). Interestingly, based on our RACE-PCR results, we found that the N-terminal end of the gene *Zt80707* encodes a predicted signal peptide transcribed only in *Z*. *tritici*. We computationally predicted the signal peptide with low probability scores using SignalP v3 [14]. Only the C score (the raw cleavage site score recognizing signal peptide cleavage sites) supported the presence of a signal peptide at the N-terminal end of the gene *Zt80707* (maximal value of C score = 0.581). This weakly predicted signal peptide corresponds exactly to the upstream sequence that is not transcribed in *Z*. *pseudotritici* or *Z*. *ardabiliae*. To experimentally confirm that this signal peptide is transcribed in *Zt80707* and targets the translated protein for secretion, we designed an *in vitro* secretion assay. We used quantitative PCR to measure the expression of *Zt80707* and its orthologs in *Z*. *pseudotritici* and *Z*. *ardabiliae in vitro*, and found that the gene is only weakly expressed during axenic growth. Therefore, the constitutive glyceraldehyde-3-phosphate dehydrogenase (gpdA) promoter from *Aspergillus nidulans* [15] was used to express *Zt80707* and *Zp80707 in vitro* in *Z*. *tritici* cells. Furthermore, we fused both genes with a C-terminal green fluorescent protein (GFP) tag. As a positive control for protein secretion, we used the well-characterized Lysine motif (LysM) effector protein Zt111221 [16,17] and as a negative control, we used the non-secreted protein Zt77228 (a predicted member of the intramitochondrial-sorting protein family). The two genes encoding Zt111221 and Zt77228 were expressed as *Zt80707* and *Zp80707* with a C-terminal GFP tag under the control of the gpdA promoter. Western blot analyses confirmed that Zt80707 is present in both the pellet and supernatant fraction of axenically grown *Z*. *tritici* cells, as also shown for the secreted LysM-positive control (Fig 2). On the other hand, the orthologous protein from *Z*. *pseudotritici* and the non-secreted negative control Zt77228 were only detectable in the pellet fraction, revealing that the *Z*. *pseudotritici* ortholog is not secreted. Together, these results supported an extracellular function of the protein uniquely in *Z*. *tritici*.

### Reconstruction of protein structure

To link signatures of positive selection to protein structure and function, we applied a computational method for protein structure prediction; I-TASSER (Iterative Threading ASSEmbly Refinement) [18]. Due to the absence of any homologous entries for Zt80707, Zt103264 and Zt110804 in the relevant databases it was not possible to predict the structures of the proteins encoded by these three genes. However, the structural conservation of Zt89160 allowed us to predict the protein structure (S5 Fig). Homologs of Zt89160 can be found in other Dothideomycete fungi, however a pathogenicity-related function has never been shown. The predicted structure of Zt89160 resembles a ring-like RCC1 β-propeller structure containing multiple lateral loops [10]. We related the predicted structure of Zt89160 to a well characterized RCC1 protein of *Drosophila melanogaster* known to interact with nucleosomes (S5 Fig) [10]. The conserved structure of the predicted RCC1 protein in *Z*. *tritici* supports a similar DNA or protein-binding function as described for other RCC1 proteins. Based on the structure of the protein we could assign each site to either central or surface regions. In general we find an excess of substitutions on the surface of the Zt89160 protein; 15.6% in surface regions vs. 4.8% in central regions (Fisher’s exact test, p-value = 0.00422). This effect is essentially due to an excess of non-synonymous substitutions; 61.1% of all non-synonymous substitutions are in surface regions vs. 38.9% in central regions (Fisher’s exact test, p-value = 0.000405), compared to 20% of all synonymous substitutions in surface regions vs. 80% in central regions (Fishers’ exact test, p-value = 1) (S5 Fig). The fact that majorities of amino acid differences between *Zymoseptoria* species locate on the surface of the protein suggests that positive selection was driven by divergent DNA sequences or proteins interacting with the different RCC orthologs in *Z*. *tritici*, *Z*. *pseudotritici* and *Z*. *ardabiliae*.

### *Zt80707*, *Zt89160*, *Zt103264* and *Zt110804* are differentially expressed during hemibiotrophic growth

**Fig 3:**
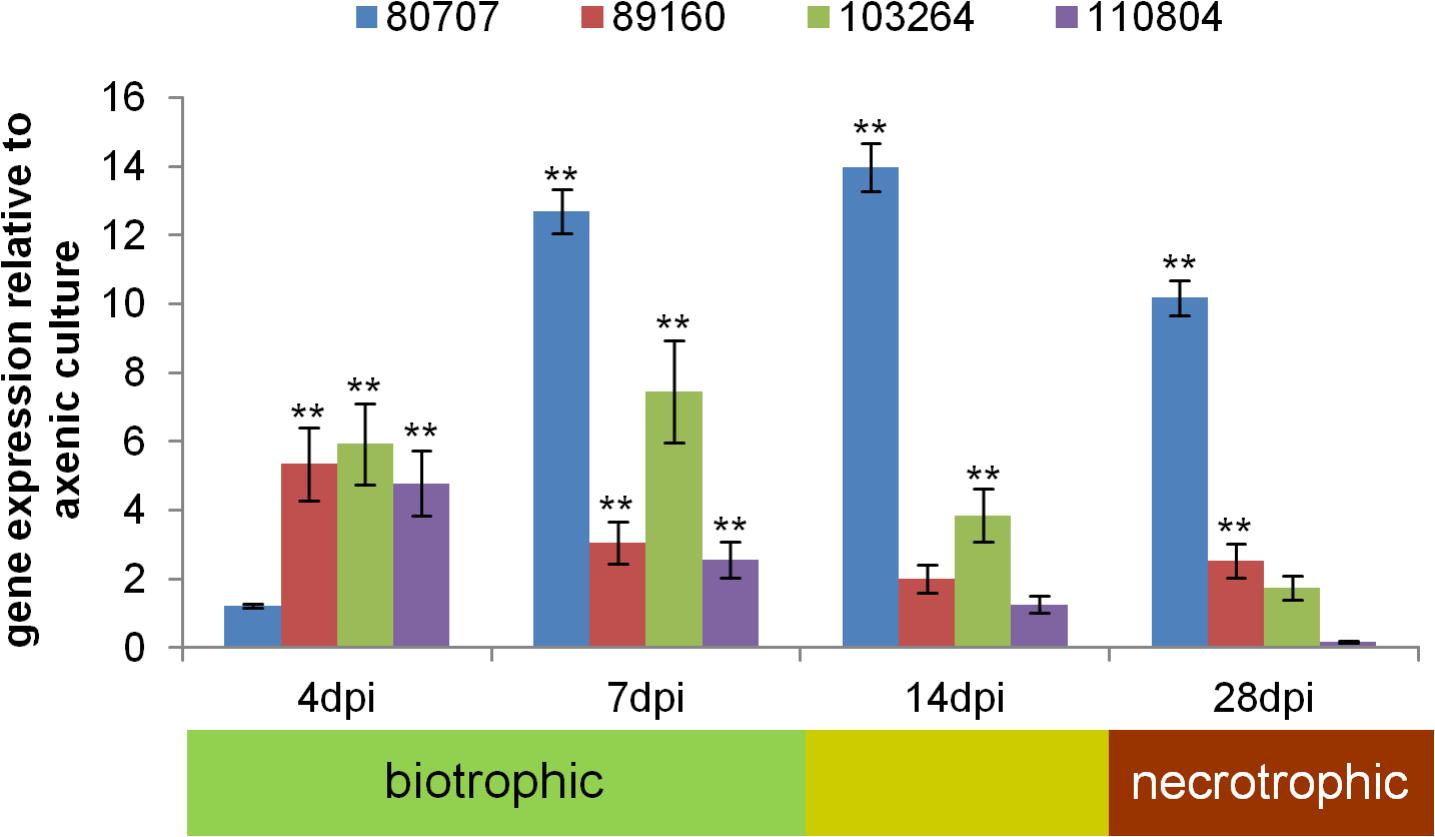
The four positively selected candidate genes are expressed during host infection. *In planta* expression data for the four positively selected candidate genes *Zt80707*, *Zt89160*, *Zt103264* and *Zt110804* of *Z*. *tritici*, relative to expression during axenic growth. Values are normalized to the expression of the gene encoding GAPDH, a constitutively expressed housekeeping control. Error bars indicate the standard error of the mean (SEM) of three independent biological replicates per sample (** *p*<0.01; *p*-values were calculated using the Mann-Whitney U-test).

To assess the expression of *Zt80707*, *Zt89160*, *Zt103264* and *Zt110804 in planta*, we performed a quantitative RT-PCR (qRT-PCR) experiment. RNA was extracted from *Z*. *tritici* from axenic cultures and from infected wheat leaves at 4, 7, 14 and 28 days post infection (dpi). These time points correspond to initial infection (4 dpi), biotrophic growth (7 dpi), a metabolic switch from biotrophic to necrotrophic growth (14 dpi) and necrotrophic growth (28 dpi). In general, all four genes were upregulated in wheat seedlings during the entire infection cycle, indicating that their function relates to *in-planta* growth (Fig 3). However, the four genes exhibited different expression patterns during biotrophic and necrotrophic growth and may be involved in distinct processes during development of infectious hyphae *in planta*. *Zt80707* is upregulated during all phases of infection except for the initial infection stage at 4 dpi. The three genes *Zt89160*, *Zt103264* and *Zt110804* show the highest expression during biotrophic growth.

### Deletion of candidate genes

To investigate the functional role of *Zt80707*, *Zt89160*, *Zt103264* and *Zt110804*, we generated independent deletion mutants in the *Z*. *tritici* isolate IPO323 using an *Agrobacterium tumefaciens* mediated transformation (ATMT) approach [16]. We verified the correct integration of hygromycin deletion constructs by homologous recombination with Southern blot analyses (S6 Fig).

**Fig 4:**
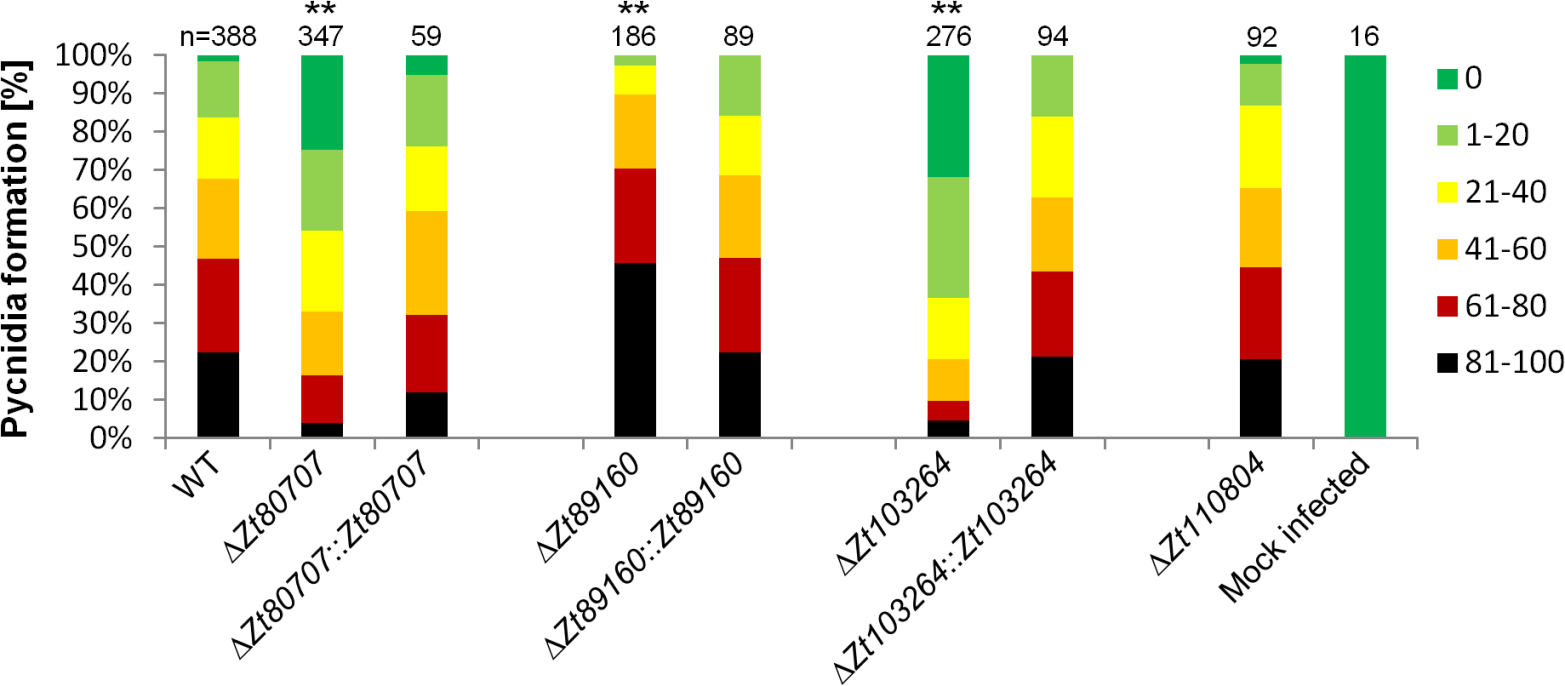
*In planta* phenotypic assay demonstrates impact of gene deletion in *Z*. *tritici*. Mutant phenotypes of *Z*. *tritici* are characterized by measures of leaf area covered with pycnidia on the susceptible wheat variety Obelisk. To compare quantitative differences in pycnidia levels at 28 dpi, six categories ranging from 0 (without any visible symptoms), 1 (1–20%), 2 (21–40%), 3 (41–60%), 4 (61–80%) and 5 (81–100%) are used. Significant deviations in pycnidia levels between the wild-type strain and mutants are observed for the IPO323Δ*Zt80707*, IPO323Δ*Zt89160* and IPO323Δ*Zt103264* mutants (** p<0.01), but not the IPO323Δ*Zt110804* mutant. Wild-type pycnidia levels for IPO323Δ*Zt80707*, IPO323Δ*Zt89160* and IPO323Δ*Zt103264* are restored by insertion of the wild-type gene at the respective native loci in the mutants. P-values were calculated using the Mann-Whitney-U-Test.

To determine any putative non-pathogenicity-related functional role we first conducted an *in vitro* phenotypic characterization of the deletion mutants. For each gene, four independent deletion strains were used in an *in vitro* stress assay using NaCl (1.5 M), H_2_O_2_ (2 mM), Congored (500 μg/ml), Calcofluor (200 μg/ml) and 28°C temperature stress. We found no difference in the sensitivity to osmotic, oxidative and cell wall stresses between the wild-type and the four deletion mutants, further supporting an *in-planta* related role of the genes (S7 Fig). Next, we performed plant experiments to determine the pathogenicity of the *Z*. *tritici* deletion mutants on the susceptible wheat variety Obelisk (Wiersum Plantbreeding, Winschoten, Netherlands). Disease development was evaluated 28 dpi by assessing the percentage of the leaf area covered with asexual fruiting bodies (pycnidia) of the *Z*. *tritici* strains (Figs 4 and S8). The formation of pycnidia was significantly reduced on leaves infected with the IPO323Δ*Zt80707* and IPO323Δ*Zt103264* mutants. Interestingly, the IPO323Δ*Zt89160* mutant caused a significantly higher amount of pycnidia, consistent with a hypervirulent phenotype. Pycnidia formation was however not affected in the IPO323Δ*Zt110804* mutant. To verify that the observed phenotypic differences were solely caused by the deletion of our candidate genes, we reintroduced the *Zt80707*, *Zt89160* and *Zt103264* open reading frames (ORFs) into the deletion strains at the endogenous locus of each gene. We confirmed that the inserted wild-type genes were all expressed as wild-type during early host infection using qRT-PCR (S9 Fig). Plant infections of these complementation strains showed that wild-type virulence could be restored for all three genes by re-insertion of the respective wild-type genes at their native locus and confirm a virulence-related role of *Zt80707*, *Zt89160* and *Zt103264* in *Z*. *tritici* (Fig 4).

### Replacement of *Z*. *tritici* genes with the orthologous genes of *Z*. *pseudotritici* and *Z*. *ardabiliae*

We hypothesize that positively selected amino acid changes in *Z*. *tritici* have played a role during speciation and adaptation to the wheat host. To test the importance of species-specific substitutions in the three candidate genes *Zt80707*, *Zt89160* and *Zt103264*, we replaced them with the respective orthologous genes from *Z*. *pseudotritici* and *Z*. *ardabiliae*. We confirmed that the inserted genes, still under the control of the native *Z*. *tritici* promoters, were expressed as in wild-type using qRT-PCR (S9 Fig).

**Fig 5:**
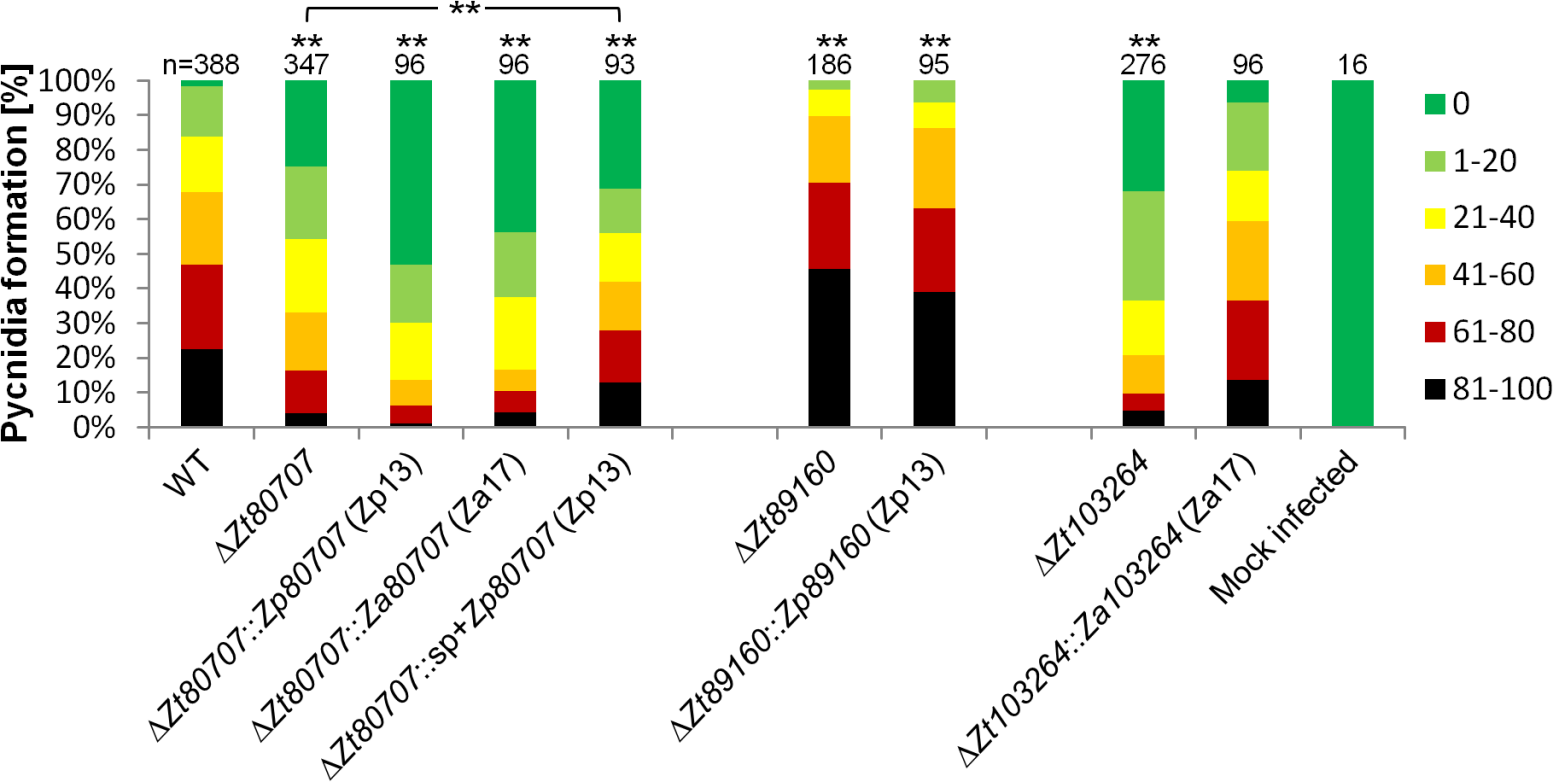
*In planta* phenotypic assay demonstrates impact of gene replacement in *Z*. *tritici*. To assess the functional relevance of non-synonymous changes in *Zt80707*, *Zt89160* and *Zt103264*, the orthologous genes from *Z*. *pseudotritici* and *Z*. *ardabiliae* were inserted at the respective native loci in the deletion mutants IPO323Δ*Zt80707*, IPO323Δ*Zt89160* and IPO323Δ*Zt103264*. For the gene *Zt80707*, the *Zp80707* ortholog was inserted with (IPO323Δ*Zt80707*::*sp*+*Zp80707)* and without (IPO323Δ*Zt80707*::*Zp80707*) the signal peptide of *Zt80707*. Quantitative differences in pycnidia levels were measured according to six categories: 0 (without any visible symptoms), 1 (1–20%), 2 (21–40%), 3 (41–60%), 4 (61–80%) and 5 (81–100%). Significant differences in pycnidia levels (** *p*<0.01) were documented using a Mann-Whitney-U-Test for the *Zt80707* and *Zt89160* mutants. Only the replacement strain IPO323Δ*Zt103264*::*Za103264* could restore the wild-type phenotype of the *Z*. *tritici* isolate IPO323.

For *Zt80707*, we replaced the gene in *Z*. *tritici* with either the full-length ortholog from *Z*. *pseudotritici* (Zp13) *Zp80707* or the full-length ortholog from *Z*. *ardabiliae* (Za17). The 3’ end of *Za80707* in *Z*. *ardabiliae* is 21 aa shorter than the orthologs of *Z*. *tritici* and *Z*. *pseudotritici* (Fig 1). The replacement with both the *Z*. *ardabiliae* and *Z*. *pseudotritici* orthologs in *Z*. *tritici* thereby also allowed us to assess the importance of the different transcription stop sites for virulence of *Z*. *tritici* on wheat. Furthermore, we generated a fusion construct with the *Zt80707* signal peptide (until the cleavage site at aa position 21) and the ORF of the Zp13 ortholog (S10 Fig). Our aim was to assess whether the *Z*. *pseudotritici* ortholog could complement the *Z*. *tritici* gene if secreted as the native *Z*. *tritici* protein. Replacing the *Z*. *tritici* genes with orthologs from *Z*. *pseudotritici* or *Z*. *ardabiliae* showed that wild-type virulence could not be restored in any of the replacement strains (Fig 5). However, with the fusion construct of the *Zt80707* signal peptide and the Zp13 ORF, it was possible to partially restore wild-type virulence levels, since the resulting amount of pycnidia produced by the mutant Δ*Zt80707*::sp+*Zp80707* was higher than pycnidia produced by the deletion strain IPO323Δ*Zt80707*. This result suggests that the protein encoded by *Zt80707* plays an essential role in the extracellular space of *Z*. *tritici* and that the amino acid substitutions in Zp80707, acquired since the divergence of *Z*. *tritici* and *Z*. *pseudotritici*, to some extent allow the protein to fulfill the same function as Zt80707.

Replacement of *Zt89160* with the orthologous gene from *Z*. *pseudotritici* (isolate Zp13) could not restore wild-type virulence in *Z*. *tritici* (Fig 5). The replacement strain showed the same hyper-virulent phenotype as the mutant, suggesting that adaptive substitutions in *Zt89160* indeed have been important for the divergent specialization of *Z*. *tritici*. The deletion of *Zt103264* was replaced with the ortholog of *Z*. *ardabiliae* (Za17). The introduction of this ortholog could, in contrast to the orthologs of *Zt80707* and *Zt89160*, restore virulence levels of the wild-type *Z*. *tritici* isolate, suggesting that adaptive substitutions in this gene do not directly relate to host specialization in *Z*. *tritici*.

### IPO323Δ*Zt80707* and IPO323Δ*Zt103264* mutants are impaired in pycnidia maturation

**Fig 6:**
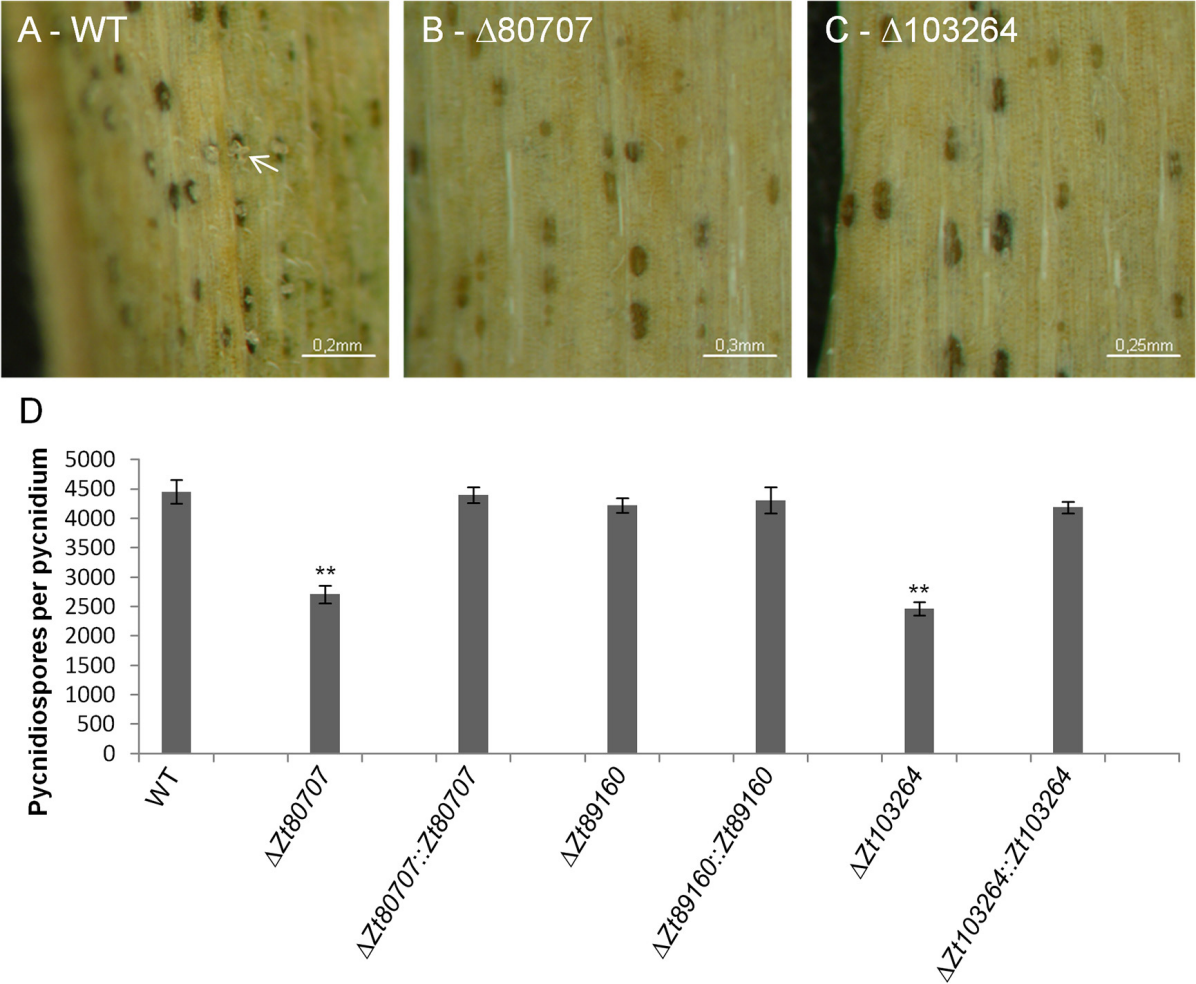
Mutant IPO323Δ*Zt80707* and IPO323Δ*Zt103264* produce fewer pycnidiospores per pycnidium. Macroscopic images of infected wheat leaves 28 dpi with wild-type IPO323 (A) and the two mutants IPO323Δ*Zt80707* (B) and IPO323Δ*Zt103264* (C). Pycnidiospores are oozing from the pycnidia of IPO323 (A, white arrow), but not from the pycnidia of the two mutants. D) A quantitative comparison of pycnidiospores produced by the wild-type and the three mutant strains IPO323Δ*Zt80707*, IPO323Δ*Zt89160* and IPO323Δ*Zt103264* measured as counts of spores isolated from a known number of pycnidia. ** *p*<0.01; *p*-values were calculated using a Mann-Whitney U test. Whiskers indicate standard deviations.

As shown in Fig 4, the IPO323Δ*Zt80707* and IPO323Δ*Zt103264* deletion mutants caused reduced amounts of pycnidia on wheat leaves 28 dpi. To further understand the impact on pycnidia production of gene products of these two genes and *Zt89160*, we conducted a more detailed comparison of pycnidia development and size. We first of all observed a delayed development of pycnidia and a delayed release of pycnidia spores in the two deletion mutants IPO323Δ*Zt80707* and IPO323Δ*Zt103264* compared to the wild-type strain. Twenty-eight dpi pycnidiospores were exuded from pycnidia on wild-type-infected leaves, but not from mutant-infected leaves (Fig 6A–6C). To evaluate the viability of these two mutants pycnidiospores, we conducted a qualitative assay comparing the pycnidia from leaf samples infected with the wild-type, the IPO323Δ*Zt89160* mutant and the two deletion mutants impaired in pycnidia production. We harvested infected leaves and induced oozing (release of spores) from pycnidia under high-humidity conditions (S11A Fig). Pycnidiospores were released from pycnidia after seven days. To assess and compare the viability of spores of wild-type and mutant pycnidia, we isolated spores from each leaf sample and prepared a dilution series to determine the proportions of germinating spores (S11B Fig). We observed no significant difference in the viability of spores from wild-type and the three deletion mutants, suggesting that there is no qualitative effect of the gene deletion of *Zt80707* or *Zt103264* on pycnidiospores.

To further investigate the quantitative differences in pycnidiospore production between deletion mutants of *Zt80707*, *Zt89160* and *Zt103264* and wild-type that we observe macroscopically, we assessed the amount of pycnidiospores per pycnidium. To do so, we also induced oozing from pycnidia on harvested leaves. We first counted the number of pycnidia per leaf, and then used a “Neubauer-improved” counting chamber to count the amount of pycnidiospores isolated from the oozing pycnidia. The number of pycnidiospores in the spore suspensions was divided by the number of pycnidia on each leaf from which the spores were isolated. This process enabled us to calculate the number of pycnidiospores per pycnidium. Our findings showed that the pycnidia of the two deletion strains IPO323Δ*Zt80707* and IPO323Δ*Zt103264* contained significantly fewer pycnidiospores than the wild-type pycnidia (Fig 6D). However, for the hypervirulent mutant IPO323Δ*Zt89160* we observed no quantitative difference of pycnidiospores compared to wild-type. The effect on pycnidiospore production could be restored for both IPO323Δ*Zt80707* and IPO323Δ*Zt103264* mutants by reintroducing the respective gene into the deletion strain (Fig 6D).

**Fig 7:**
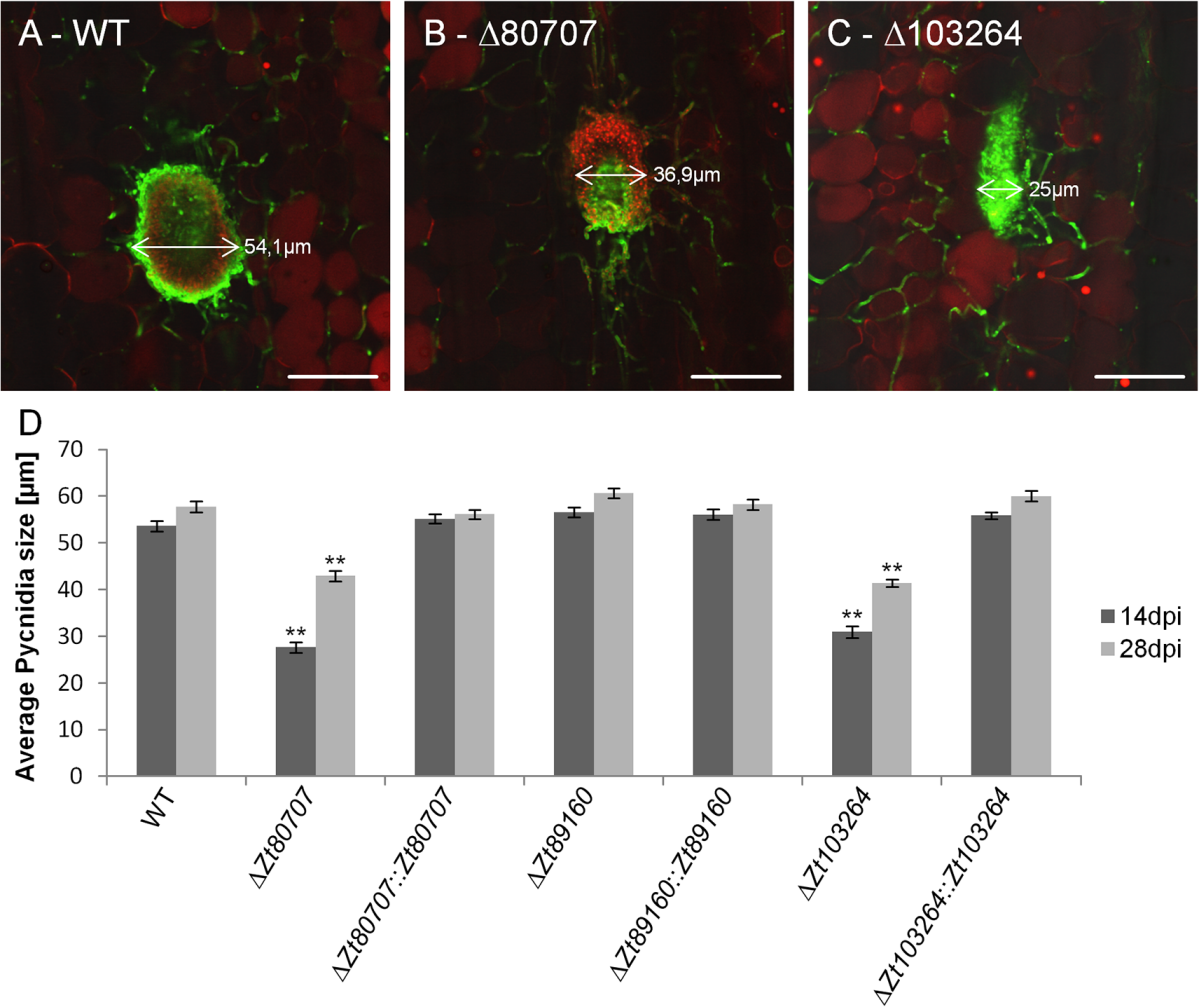
Confocal laser scanning microscopy image analyses of pycnidia of wild and mutant *Z*. *tritici*. WGA-FITC / Propidium iodide double-stained pycnidia on infected wheat leaves 28 dpi of wild-type (A), mutant *Z*. *tritici* isolates; IPO323Δ*Zt80707* (B) and IPO323Δ*Zt103264* (C). Fungal cell walls (green) and fungal and plant nuclei (red) are visible in the infected plant tissue (scale bar: 50 μm). D) Average size of pycnidia measured 14 and 28 dpi. For each strain and time point, 50 pycnidia were measured. ** *p*<0.01; *p*-values were calculated using a Mann-Whitney U test. Whiskers indicate standard deviations.

We next confirmed the developmental defect of the pycnidia formation in IPO323Δ*Zt80707* and IPO323Δ*Zt103264* in comparison to wild-type and the hypervirulent mutant IPO323Δ*Zt89160* using confocal microscopy. We stained infected leaf samples at 14 and 28 dpi using a wheat germ agglutinin—fluorescein isothiocyanate / propidium iodide double staining to visualize both the fungus and plant cells. We measured the width of the pycnidia (n = 50 for each strain and time point) on wheat leaves infected by the wild-type strain and mutants. We find that the size of the pycnidia generated by wild-type *Z*. *tritici* and the mutant IPO323Δ*Zt89160* is almost unchanged from 14 dpi to 28 dpi at 50–60 μm (Fig 7). Furthermore, we found that the mean pycnidia size of IPO323Δ*Zt80707* and IPO323Δ*Zt103264* is significantly smaller than the wild-type pycnidia at both time points of infection, 14 and 28 dpi (Fig 7). Pycnidia produced by the hypervirulent mutant IPO323Δ*Zt89160* on the other hand did not deviate from wild-type pycnidia. We confirmed that the effect found in the IPO323Δ*Zt80707* and IPO323Δ*Zt103264* mutants is due to the deletion of the two genes, since reintroduction of the respective genes could restore wild-type pycnidia development.

## Discussion

Recent speciation of the wheat pathogen *Z*. *tritici* entailed adaptation to a new host and involved a strong effect of natural selection during divergence from a common ancestor of *Z*. *tritici*, *Z*. *pseudotritici* and *Z*. *ardabiliae* [8]. We hypothesize that signatures of positive selection in the genomes of these pathogens reflect those traits that have been important for divergent host specialization. The functional analyses of four positively selected genes in *Z*. *tritici* allowed us to identify three genes with significant impact on disease development in wheat. For two of these three genes, *Zt80707* and *Zt103264*, we could furthermore show that *Z*. *tritici*-specific amino acid changes are crucial for virulence in wheat. Rapid evolution and positive selection of pathogenicity related genes have been documented in other filamentous plant pathogens [5,19,20]. Aguileta and colleagues used genome data and EST libraries from different species of *Botrytis* and *Sclerotinia* to search for genes with signatures of positive selection in a dataset of 642 orthologs [21]. Of 21 positively selected genes, four genes were further tested for virulence related functions, but proved non-essential for disease development of *Botrytis* at least as tested under laboratory conditions. The four genes studied by Aguileta and colleagues and the gene Zt110804 included in our functional analyses demonstrate that the relevance of positively selected amino acid changes cannot always be detected with standard experimental assays. Other functional assays could include a variety of host genotypes or different environmental conditions to determine eventual fitness effects in mutant strains.

For two of the genes studied here, *Zt80707* and *Zt10324*, we not only find variation at the level of nucleotide substitutions, but also at the level of reading frame structure. To our knowledge this is the first report of large variation of reading frame structure variation in virulence related genes of a fungal plant pathogen. A dramatic consequence of the different transcript start of *Zt80707* in *Z*. *tritici* is a signal peptide that is only transcribed and translated in *Z*. *tritici*. Thus, in *Z*. *tritici*, the protein encoded by *Zt80707* acts in the extracellular space and may interact with host-produced proteins while orthologous proteins in *Z*. *pseudotritici* and *Z*. *ardabiliae* act intracellularly. In *Z*. *ardabiliae*, but not in *Z*. *pseudotritici*, there is a methionine at the corresponding site of the transcription start in *Z*. *tritici*. However, our 5’ RACE-PCR experiment clearly shows that the transcription start of *Za80707* is initiated 25 amino acids downstream (S4 Fig). Either, transcription of the signal peptide in *Z*. *pseudotritici* and *Z*. *ardabiliae* was lost after divergence of the *Z*. *tritici* lineage, or, more plausible, the earlier transcription start originated more recently in the *Z*. *tritici* lineage. We speculate that non-synonymous substitutions in the *Z*. *tritici* gene relate to a novel function of the protein in the extra-cellular space. Indeed the function of Zt80707 could not be fully restored by integration of the orthologs from *Z*. *pseudotritici* and *Z*. *ardabiliae* with or without the secretion signal (Fig 5). Further characterization of protein function in *Z*. *pseudotritici*, *Z*. *ardabiliae* and the more distantly related sister species *Z*. *passerinii*, will be necessary to clarify the ancestral structure and function of the protein.

The deletion mutants of both *Zt80707* and *Zt103264* are still able to infect and reproduce asexually in wheat, however the mutants are significantly impaired in the development of pycnidia. We measured this as a reduced number of pycnidia and as significantly smaller pycnidia in the mutant-infected plants. *Zt103264* is upregulated during early host colonization and may therefore play a role in the early establishment of biotrophic growth and defeat of host defenses. The observed effect on pycnidia production would thereby be a secondary effect following lower biomass and impaired pathogen development. *Zt80707* is expressed during necrotrophic growth and pycnidia formation. In other ascomycete fungi, asexual spore formation has been linked to primary and secondary metabolite production [22, 23]. Consistent with an extracellular function of Zt80707 in *Z*. *tritici*, we did not detect any difference in pigment production during an *in vitro* cell wall and temperature stress assay. Nor did we observe differences in the growth morphology of yeast-like cells or hyphae. We speculate that Zt80707 instead plays a role in host-fungus signaling and interaction and indirectly affects the development of pycnidia in *Z*. *tritici*.

The quantitative impact on disease development by the two mutants IPO323Δ*Zt80707* and IPO323Δ*Zt103264* suggest that multiple gene products contribute to virulence in *Z*. *tritici*. A single determining virulence factor has also been described in *Z*. *tritici* [16]. Deletion of the Mg3LysM effector causes full virulence defect in *Z*. *tritici*. LysM effectors are known to interfere with chitin-triggered immunity in plants and have been described in a number of fungal plant pathogens [17]. It is likely that *Z*. *tritici* encodes an arsenal of effector proteins during early biotrophic colonization of wheat. These genes may also evolve by positive selection driven by an antagonistic arms-race evolution between effectors and their target proteins. However, the genes picked up by our comparative genome analyses and further investigated here reflect signatures of past positive selection related to speciation and divergent host specialization. Sequencing of more *Z*. *tritici* genomes will allow inference of ongoing positive selection in the genome of the wheat pathogen.

Our third candidate, *Zt89160*, exhibited a hypervirulent phenotype. Mutant hypervirulence has only been described in a few examples of fungal plant pathogens [24–27]. Zt89160 contains two RCC1-like domains. RCC homologues have been characterized as nuclear proteins in many eukaryotes, including *Saccharomyces cerevisiae* and *Schizosaccharomyces pombe* [28–31]. Disruption of the gene in different organisms affects RNA processing and transport, mating, initiation of mitosis and chromatin condensation. So far RCC1 proteins have never been studied in a fungal plant pathogen or connected to fungal pathogenicity. We speculate that Zt89160 could play a central role in the regulation of virulence-related genes in *Z*. *tritici* consistent with the increased pycnidia formation in the deletion mutant. The hemibiotrophic nature of *Z*. *tritici* requires a fine-tuned regulation of transcription during host infection and the switch from biotrophic feeding to necrotrophic feeding, and this regulation may be affected in the IPO323Δ*Zt89160* mutant. Positive selection acting on the gene could reflect changes in the binding sites of Zt89160 relative to the orthologs in *Z*. *pseudotritici* and *Z*. *ardabiliae*. This hypothesis is supported by the fact that most amino acid substitutions locate on the outer surface of the protein (S5 Fig).

In summary, we here show a strong correlation between evolutionary predictions and virulence function in a plant-pathogenic fungus. Previous studies of prokaryote and eukaryote pathogenic species have likewise demonstrated accelerated evolution of virulence-related genes [3,32]. The focus of these studies has been the evolution of effector-encoding genes, typically small, secreted proteins. Our selection of candidate genes has however been determined only on the basis of evolutionary predictions and no *a priori* information about gene function or structure. We demonstrate that adaptive evolution during host specialization also strongly affects non-secreted proteins without a putative effector function (Zt89160, Zt103264 and Zt110804). Even so, several of these genes may play a central role in virulence through the regulation of other genes or impact on *in planta* development of hyphae and spore production. The findings presented here suggest that the integration of evolutionary predictions and functional analyses provide a strong framework for the identification of new pathogenicity-related traits and host species determinants of pathogens.

## Materials and Methods

### Positively selected candidate genes

The four genes, *Zt80707*, *Zt89160*, *Zt103264* and *Zt110804*, were selected according to their elevated rates of non-synonymous substitutions in *Zymoseptoria tritici*. Signatures of strong positive selection in these four genes were previously demonstrated using the approach of Nei and Gojobori [9] in a comparative genome study, including full genome sequences of *Z*. *tritici* and two closely related species, *Z*. *pseudotritici* and *Z*. *ardabiliae* [8]. The genomic coordinates of the candidate genes in the *Z*. *tritici* reference genome [33] are provided in Table 1.

### Validation of gene structures by RACE-PCR

To validate gene structure, including start and stop codons and intron-exon boundaries, we conducted 5'-RACE-PCR in *Z*. *tritici*, *Z*. *pseudotritici* and *Z*. *ardabiliae* for the genes *Zt80707* and *Zt103264* and their orthologs in *Z*. *pseudotritici* and *Z*. *ardabiliae*. RNA extraction was conducted as described below from axenic cultures grown in YMS medium from the *Z*. *tritici* isolate IPO323, the *Z*. *pseudotritici* isolate STIR04_2.2.1 (Zp13) and the *Z*. *ardabiliae* isolate STIR04_1.1.1 (Za17) [8]. We used the 5’ RACE System for Rapid Amplification of cDNA Ends Kit (Invitrogen, Karlsruhe, Germany) and designed three gene-specific primers (GSPs) for each gene for the first strand cDNA synthesis, for a first PCR GSP2 and a nested GSP3 for a nested PCR (S1 Table). The resulting PCR products were cloned into the TOPO (Invitrogen, Karlsruhe, Germany) backbone and subsequently sequenced.

### Analyses of positive selection in candidate genes

Gene sequences were aligned in Seaview [34] using the program muscle [35]. We re-analyzed gene alignments of *Zt80707*, *Zt89160*, *Zt103264* and *Zt110804* to assess *d*_N_/*d*_S_ ratios (the non-synonymous substitutions rate divided by the synonymous substitutions rate) using the codeml program from the Phylogenetic Analysis by Maximum Likelihood (PAML) package [36]. The approach used by Nei and Gojobori allowed us to identify an excess of non-synonymous mutations; however, it did not provide information about the non-homogenous rates of evolution among the three *Zymoseptoria* species. With the new gene alignments, we asked whether any of the four genes in particular had experienced accelerated evolution in the wheat pathogen *Z*. *tritici*. Using the program PhyML [37], we generated a phylogenetic tree for each gene alignment, including sequence data from the more distantly related species *Z*. *passerinii*. We calculated *d*_N_/*d*_S_ ratios for the individual branches of the trees [36]. Branch-specific *d*_N_/*d*_S_ ratios above 1 are indicative of positive selection.

### Prediction of the protein structures

Signal P v3 was used for prediction of secretion signals [14]. For the prediction of the protein structure we used the I-TASSER program [18]. The program is based on a composite approach of many threading (fold recognition) programs to structure alignments. The quality of the predicted protein structure is evaluated using the C-score (-0.4) and the TM-score (0.66) which both indicate an evaluation of the structure prediction. For the three protein products of *Zt80707*, *Zt103264* and *Zt110804* we were not able to obtain significant structure predictions. We could however obtain a reliable prediction for the protein structure of Zt89160. For this protein, we determined the surface accessible amino acids using the SwissPDB-Viewer [38]. The protein structure was visualized using the PyMOL Molecular Graphics System [39].

### Fungal and bacterial strains

For all *in planta* and transformation experiments, we used the reference isolate of *Z*. *tritici* IPO323 [40]. The genome sequence of IPO323 is available from http://genome.jgi-psf.org/Mycgr3/Mycgr3.home.html [33]. The *Z*. *tritici* gene IDs that we used here correspond to the Mgr gene IDs used in the JGI genome database. Furthermore, we used the *Z*. *pseudotritici* isolate ST04IR_2.2.1 (Zp13) and the *Z*. *ardabiliae* isolate ST04IR_1.1.1 (Za17), for which genome sequences are available from the National Center for Biotechnology Information (NCBI) database (taxonomy ID: 985140 and 985147, respectively) [8]. The isolates were inoculated from glycerol stocks onto solid yeast-malt-sucrose agar plates at 18°C. Yeast-like cells grown on these plates were used as inoculum for all experiments. All plasmids used in our study were maintained in *E*. *coli* Top10 cells (Invitrogen, Karlsruhe, Germany). The *Agrobacterium tumefaciens* strain AGL1 was used for *Agrobacterium tumefaciens* mediated transformation (ATMT) of the fungal cells. Both bacterial strains were grown on double yeast tryptone (dYT) medium. For the maintenance of the plasmids already present in the AGL1 strain, 50 μg/ml Rifampicin (Sigma, Taufkirchen, Germany) and 100 μg/ml Carbenicillin (Sigma, Taufkirchen, Germany) were added to the dYT medium.

### ATMT of fungal cells

To assess the functional role of the four candidate genes, *Zt80707*, *Zt89160*, *Zt103264* and *Zt110804*, we generated four deletion mutants in *Z*. *tritici*. For targeted gene deletion, we amplified a DNA fragment for each gene, including the ORF and 1 kb upstream and downstream sequence by PCR. Each amplification product was fused with a hygromycin resistance cassette [41] using an overlap PCR approach [42]. Deletion constructs were ligated into the pES22 plasmid via two restriction sites added by outer primers (S6 Fig and S1 Table). The plasmid pES22 is a derivate of the binary vector pNOV-ABCD previously developed for targeted gene deletion in *Z*. *tritici* [41]. We furthermore generated constructs for complementation tests of *Zt80707*, *Zt89160* and *Zt103264*. For each construct, a DNA fragment, including the coding sequence of the candidate gene and 1 kb of upstream and downstream sequence, was fused together with a Geneticin (G418) resistance, cloned into the pES22 plasmid cassette using Gibson assembly [43] and introduced into the respective deletion strain. The same approach was conducted to replace the *Z*. *tritici* genes with orthologs from *Z*. *pseudotritici* or *Z*. *ardabiliae*. As for the complementation strains, orthologous genes were fused in frame to the respective *Z*. *tritici* promoter and introduced in the deletion mutants using ATMT. In total we generated five replacement strains including IPO323Δ*Zt80707*::sp+*Zp80707* IPO323Δ*Zt80707*::*Zp80707* IPO323Δ*Zt80707*::*Za80707*, IPO323Δ*Zt89160*::*Zp89160*, IPO323Δ*Zt103264*::*Za103264*. Electro-competent cells of the *A*. *tumefaciens* strain AGL1 were transformed with the final plasmids (S2 Table) using standard procedures. For ATMT of *Z*. *tritici*, we used the protocol described by Zwiers and De Waard (2001) [44]. Transformed fungal colonies were visible on hygromycin- or Geneticin-containing plates two weeks after transformation. Colonies obtained from single cells were propagated in YMS medium for further DNA extraction, PCR and Southern blot analysis.

### Isolation of fungal genomic DNA and Southern blot analysis

Fungal DNA was extracted using a standard phenol-chloroform extraction protocol [45]. We first screened transformed strains using a PCR-based approach, amplifying the hygromycin resistance cassette and the endogenous locus using the outer primers of the deletion constructs (S1 Table and S6 Fig). To confirm homologous recombination and correct transformation, we performed a Southern blot analysis using standard procedures [46]. Probes were generated using the PCR Digoxigenin (DIG) Labeling Mix (Roche, Mannheim, Germany) according to the manufacturer’s instructions.

### Plant infections

For plant infections, we used 15-day-old wheat (*Triticum aestivum*) seedlings of the cultivar Obelisk (Wiersum Plantbreeding). A spore solution of 1 × 10^7^ cells/ml containing 0.1% Tween 20 (Roth, Karlsruhe, Germany) was brushed onto an 8–10 cm marked area of the second leaf of each plant. After an initial 48-h incubation period at 100% humidity, the infected plants were incubated at 22°C with a 16-h light period at 75% humidity for another 26 days. For all experiments two independent strains (biological replicates) were used.

### Phenotypic assays

We performed an *in vitro* assay of wild-type and deletion mutants to test whether any observed phenotype relates to the host-pathogen interaction or to basic growth performance of the mutants. First, we investigated single cells of the wild-type and the deletion mutants microscopically using a light microscope (Leica DM750, Wetzlar, Germany). Next, we conducted a stress assay to investigate whether deletion mutants were affected in their response to cell stress reagents. Four μl of a spore suspension containing 1 × 10^7^ spores/ml and 6 1:10 dilutions of a dilution series were pipetted on stress plates and incubated for six days at 18°C. We grew fungal cells on plates containing NaCl (1.5 M), H_2_O_2_ (2 mM), Congored (500 μg/ml) and Calcofluor (200 μg/ml) to compare the sensitivity of strains to osmotic and oxidative cell wall stresses. We also incubated fungal colonies at 28°C to assess temperature sensitivity. Wild-type, mutant and complementation strains were all assayed for their *in vitro* phenotypes. To investigate the effect of gene deletion *in planta*, we compared disease development of wild-type and mutant-infected plant leaves 28 dpi. Symptoms recognized as pycnidia were evaluated. To quantify disease levels, we used a scoring scheme of six categories (0%, 1–20%, 21–40%, 41–60%, 61–80% and 81–100%) representing the percentage of the leaf area covered with pycnidia. 48 wheat leaves infected with wild-type were compared to 59–96 leaves infected with deletion mutants, complementation or replacement strains (Figs 4 and 5). Disease scoring was done by eye always by the same person. A Mann-Whitney-U-test was applied to test the statistical significance of the observed differences between wild-type all and mutant strains.

We evaluated and compared pycnidia spore viability from wild-type and the *Zt80707*- and *Zt103264*-deletion mutant-infected leaves. The infected leaves were harvested four weeks after infection and surface sterilized using 5% sodium hypochlorite and 70% ethanol. The infected leaves were incubated under high-humidity conditions for seven days on a metal grid in a sealed Petri dish in the phytochamber with the same light settings as the infection experiment (see above). The high-humidity conditions in the Petri dish induce the oozing of pycnidia and the release of spores. The whole-leaf samples were vortexed gently in 500 μl sterile H_2_O and 1:10 dilution series were made with three steps. Three μl of every dilution was pipetted on YMS and YMS-hygromycin plates. The proportions of germinating spores were compared between the wild-type and the deletion strains (S11 Fig).

To investigate the quantitative difference between deletion mutants of *Zt80707*, *Zt89160* and *Zt103264* and the wild-type strain, we estimated the number of pycnidiospores per pycnidium. Therefore, we also induced oozing from pycnidia on harvested leaves and then used a “Neubauer-improved” counting chamber to count the amount of pycnidiospores isolated from the oozing pycnidia. The number of pycnidiospores in the spore suspensions was divided by the number of pycnidia on each leaf from which the spores were isolated to estimate the number of pycnidiospores per pycnidium.

### WGA-FITC / Propidium iodide staining

Harvested leaf samples from two independent plant experiments were de-stained overnight (or longer) in 2 ml Eppendorf (Hamburg, Germany) tubes in 100% ethanol. Ethanol was exchanged if necessary. Next, the leaves were incubated in 10% KOH at 85°C for 5 min. The samples were then washed 3–4 times with PBS (pH 7.4) and the staining solution was added. The samples were vacuum infiltrated at 100 mbar using a cvc3000 vacuum controller (Vacuubrand, Wertheim, Germany). The staining solution was collected for reuse and the samples were de-stained in PBS and stored in the dark at 4°C. The staining solution was prepared using 20 μg/ml Propidium iodide, 10 μg/ml WGA-FITC, and 0.02% Tween 20 in 1× PBS (pH 7.4). Microscopy was conducted using a Leica SP5 confocal microscope. The filter wavelengths used for the detection were 488 nm (for FITC) and 561 nm (for Propidium iodide). The fluorophors were excited by an argon and a diode-pumped solid-state (DPSS) laser.

### RNA isolation and quantitative RT-PCR

We analyzed gene expression patterns of *Zt80707*, *Zt89160*, *Zt103264* and *Zt110804* in *Z*. *tritici* using a qRT-PCR experiment. Total RNA was extracted from fungal axenic cultures (grown for 72 h in YMS medium at 18°C and 140 rpm) and from freeze-dried leaf tissue infected with *Z*. *tritici* (4, 7, 14 and 28 dpi) using the TRIZOL reagent (Invitrogen), following the manufacturer’s instructions. Three biological replicates were sampled from axenically grown cultures and from each time point of infection. The samples were crushed in liquid nitrogen and 100 mg was used for RNA extraction and cDNA synthesis. The cDNA samples were used in a qRT-PCR experiment employing the iQ SYBR Green Supermix Kit (Bio-Rad, Munich, Germany), GSPs (S1 Table) and an annealing temperature of 59°C. PCR was conducted in a CFX96 RT-PCR Detection System (Bio-Rad) with the constitutively expressed control gene *Zt99044*, a Glyceraldehyde-3-phosphate dehydrogenase (GAPDH) [33]. A Mann-Whitney U test was applied to test the significance of different gene expression levels.

### Secretion assay for *Zt80707*

To investigate the presence of a putative signal peptide at the 5’ end of the gene *Zt80707*, we analyzed the transcribed sequence using SignalP 3.0 [14]. SignalP only returned weak evidence for a secretion signal and we therefore aimed to experimentally verify the putative signal peptide. To do this, we designed a construct for stable expression of *Zt80707* in a non-coding region of chromosome 1 (Chr1: 464436–466636) in *Z*. *tritici*. *Zt80707* is only weakly expressed in axenic culture and we therefore expressed it under the control of the constitutively induced gpdA promoter from *Aspergillus nidulans* [15]. To verify secretion in a Western blot-based experiment, we also fused a 3’ GFP tag to the *Zt80707* sequence. In addition to *Zt80707*, we also generated a construct with the ortholog *Zp80707* of *Z*. *pseudotritici*. The ORFs were cloned into the plasmid pES150 by Gibson assembly [43]. The positive and negative controls, *Zt111221* and *Zt77228*, respectively, were also expressed under control of the gpdA promoter and tagged with a 3’ GFP. The constructs furthermore included a Geneticin resistance cassette and 1 kb flanking region of the non-coding locus at chromosome 1 in order to allow for correct integration of the constructs at this site via homologous recombination. After transformation of the four constructs in IPO323, positive transformants were grown in 50 ml YMS medium at 200 rpm and 18°C for 72 h until an OD_600_ of 1. For protein extraction, the cultures were centrifuged at 10000× g for 15 min. Protein extraction from the cells was conducted using a peqGOLD TriFast kit (Peqlab, Erlangen, Germany), according to the manufacturer’s instructions. For precipitation of the proteins in the supernatant, 40 ml of each culture was lyophilized and dissolved again in 1 ml H_2_0. This same methodology was used for a TCA precipitation [45] and the resulting protein pellet was dissolved in 50 μl 1% sodium dodecyl sulfate (SDS). The protein concentrations were estimated using the Bradford reagent in order to load similar amounts on a 15% SDS gel. This was confirmed by a Coomassie staining of the SDS gels to show similar protein amounts for the pellet and supernatant lanes. Hereafter, a Western blot analysis was performed using electrophoretic transfer. Finally, the proteins of interest were detected using an a-GFP primary antibody (Roche), together with a horseradish peroxidase (HRP) linked secondary antibody (New England Biolabs, Frankfurt, Germany) and the Amersham ECL Prime Western Blotting Detection Reagent (GE Healthcare, Freiburg, Germany).

## Supporting Information

S1 Table List of primers used in the study. (DOCX)

S2 Table List of plasmids generated in the study. (DOCX)

S1 Fig Amino acid variation in protein alignments of Zt89160, Zt103264 and Zt110804. Protein alignment of Zt89160, Zt103264 and Zt110804 of different isolates of *Z*. *tritici* (Zt), *Z*. *pseudotritici* (Zp) and *Z*. *ardabiliae* (Za).
(TIF)

S2 Fig Conserved synteny in genomics regions encoding positively selected candidate genes. Schematic illustration of conserved gene order in genomic regions encoding *Zt80707*, *Zt89160*, *Zt103264* and *Zt110804* in *Z*. *tritici* (Zt09), *Z*. *pseudotritici* (Zp13) and *Z*. *ardabiliae* (Za17). The four candidate genes are shown in green, neighboring genes are shown in dark grey, inverted genes are shown in light grey and transposable elements are shown in red.
(TIF)

S3 Fig PAML analysis of positively selected candidate genes. Phylogenetic trees were made for *Z*. *tritici* genes *Zt80707* (A), *Zt89160* (B), *Zt103264* (C) and *Zt110804* (D) and the respective orthologs of *Z*. *pseudotritici*, *Z*. *ardabiliae* and *Z*. *passerinii*. A branch model was used to estimate the ω (*d*_N_/*d*_S_ ratio) for each branch. Branch-specific ω values above 1 are indicative of positive selection.
(TIF)

S4 Fig Amplification of cDNA of *Zt80707* and *Zt103264* reveals different transcript lengths in *Z*. *tritici*, *Z*. *pseudotritici* and *Z*. *ardabiliae*. Rapid Amplification of cDNA Ends (RACE-PCR) of *Zt80707* and *Zt103264* revealed the ORFs (black arrows) schematically illustrated, including 5’UTR (white rectangles) and start codon positions. The lengths of the ORFs were confirmed using the primer combinations “A” and “C” and “B” and “C” with cDNA of *Zt80707* and D-E and D-F of *Zt103264*. As template for the PCRs genomic DNA (g) of the three strains, Zt09, Zp14 and Za17 were used, as well as cDNA (c) obtained from infected wheat leaves.
(TIF)

S5 Fig Positively selected amino acids of Zt89160. A) Top view, side view and bottom view of the Zt89160 protein structure predicted with I-TASSER. Beta sheets are depicted in yellow and the accessible amino acids on the surface of the protein are shown in red. B) Top view, side view and bottom view of the structure predicted by I-TASSER for the *Drosophila melanogaster* protein CG6678, a protein homologous to Zt89160. Beta sheets are depicted in yellow and alpha helices are shown in red. The table shows that the significant majority of positively selected amino acids is located on the surface of the protein Zt89160. ** *p*<0.01. *p*-value calculated using a Fishers exact test.
(TIF)

S6 Fig Deletion construct and mutant screening in *Z*. *tritici*. Schematic illustration of the gene deletion approach in *Z*. *tritici* (example *Zt80707*). A) Gene deletion by homologous recombination using *Agrobacterium tumefaciens*-mediated transformation. B) PCR screening to identify correctly transformed strains using the primer combination oES19 and oES22. C) Correctly transformed strains were finally confirmed via Southern blot analysis using the oES19- and oES22-amplified sequence as a probe for hybridization.
(TIF)

S7 Fig *In vitro* stress assay for the evaluation of mutant phenotypes. Three independent mutants of each generated deletion and complementation strain for the genes *Zt80707*, *Zt89160* and *Zt103264* were tested under multiple abiotic stress conditions. The deletion of *Zt110804* was not complemented. The following conditions were used: heat stress (28°C), NaCl (1.5 M), Congored (500 μg/ml), H_2_O_2_ (2 mM) and Calcofluor (200 μg/ml).
(TIF)

S8 Fig Quantitative categories of pycnidia produced by *Z*. *tritici* during infection of wheat samples. Representative images for the six categories that were used to score disease levels at 28 dpi in plant assays. The number of pycnidia per leaf (ppl) is shown for every leaf. Areas encircled by red lines illustrate the proportion of necrotic areas with pycnidia.
(TIF)

S9 Fig Quantitative real time PCR of wild-type, complementation and replacement strains of *Zt80707*, *Zt89160* and *Zt103264*. Expression levels of the three genes in the respective complementation- and replacement strains at 4 dpi have been compared to wild-type *Z*. *tritici* infected leaves (WT). Values are normalized to the expression of the gene encoding GAPDH, a constitutively expressed housekeeping control. Error bars indicate the standard error of the mean (SEM) of three independent biological replicates per sample.
(TIF)

S10 Fig Schematic illustration of the fusion construct for the *Zt80707* signal peptide with the *Zp80707* ORF. The promoter (light green) and signal peptide (light grey) of *Zt80707* from *Z*. *tritici* were fused with the ORF of *Zp80707* from *Z*. *pseudotritici* (beginning of the ORF shown in dark grey) to obtain the secretion of Zp80707 in *Z*. *tritici*.
(TIF)

S11 Fig Qualitative assay of pycnidiospore viability in *Z*. *tritici*. A) Macroscopic picture of oozing pycnidia of an infected wheat leaf (IPO323 wild-type) after surface sterilization and one week of incubation in high humidity. B) Exuded fungal spores were isolated and added in different concentrations to YMS media to test the viability of wild-type and mutant spores.
(TIF)
