# Supplementary material for: Rapidly Evolving Genes Are Key Players in Host Specialization and Virulence of the Fungal Wheat Pathogen Zymoseptoria tritici (Mycosphaerella graminicola)
Source: PLoS Pathog. 2015 Jul 30;11(7):e1005055. doi: 10.1371/journal.ppat.1005055 (PMC4520584; doi:10.1371/journal.ppat.1005055)
Supplement: S1 Table — (DOCX) [file ppat.1005055.s001.docx]

**Supplementary Table 2**

| PCR primers used in this study | |  |  |  | |
| --- | --- | --- | --- | --- | --- |
| Name | Sequence 5’ -> 3’**^1^** | | | | Application**^2^** |
| oES174  oES175  oES176  oES177  oES178  oES179  oES1021  oES1022  oES199  oES200  oES786  oES787  oES883  oES884  oES885  oES886  oES865  oES866  oES867  oES868  oES869  oES870  oES871  oES872  oES873  oES874  oES875  oES876  oES973  oES974  oES975  oES976  oES978  oES979  oES705  oES706  oES654  oES655  oES656  oES657  oES1044  oES1045  oES1046  oES1047  oES1048  oES1049  oES1099  oES1100  oES1101  oES1053  oES1054  oES1055  oES1131  oES1132  oES1133  oES1134  oES1135  oES1136  oES1137  oES1138  oES1449  oES1450  oES1451  oES1452  oES1453  oES1454  oES1455  oES1456  oES1457  oES1458  oES1459  oES1460  oES1461  oES1462  oES1463  oES1464  oES1494  oES1495  oES1496  oES1497  oES1596  oES1597  oES1598  oES1599  oES1600  oES1601  oES1602  oES1603  oES1604  oES1605  oES1606  oES1607  oES1331  oES1367  oES1368  oES690  oES691  oES1130  oES8  oES1726  oES1727  oES1731  oES1732  oES202  oES43  oES121  oES859  oES46  oES124  oES998  oES996  oES997  oES999  oES1000  oES1001  oES1002 | CGATCGGCCTAGACGGCCTGATATTGAAGGAGCATTTT  CGATCGGCCGCATCGGCCTAGCAGATCTCTATTCCTTT  CGATCAAGCTTGATTCGACACGCATTGCGCAAAGGC  CGATCGGCCGTCTAGGCCATTGGAGTGCATTATTAGCG  CGATCGGCCGATGCGGCCATTCTGGGTATCCGTATAGCGTCT  CGATCGAATTCGCCATACCGACTCCTACCCTTCCTC  GTACCATCGACCACAATGAC  ATGCCTGTAGGGAACTTCAC  CGAGCTTGACGCAGAGTGTG  ACAGGCTCTGGCAGGAAC  TGTCGGCAAGGTCATTCCAG  ATGCGGCAGGTCAAGTCAAC  GCGGAGATGGCAACTATCCC  ACTTGGCCGCTTTCCATCAG  ATCATATCCGCCGCCACATC  TCTAGGCGTGCCTAAAGGAG  GATCGGCGCGCCCACTTTCAGGCTGCCATAAC  TTCAATATCAGGCCGTCTTGCATGTCTCCGTCGATTTC  TCAGATCATGCTGGGCTCATCGGCCTAGCAGATCTCTA  AATCGACGGAGACATGCAAGACGGCCTGATATTGAAGG  GAGATCTGCTAGGCCGATGAGCCCAGCATGATCTGAAC  GATCCCTGCAGGCTGCTGACGCATCAAAGTCC  GATCGAATTCTGGAAGGTGTTTCGGATGTC  TTCAATATCAGGCCGTCTGAGCTTGTGCTGCTAATTGC  TAGCTCAACAGTCATGCGATCGGCCTAGCAGATCTCTA  AATTAGCAGCACAAGCTCAGACGGCCTGATATTGAAGG  GAGATCTGCTAGGCCGATCGCATGACTGTTGAGCTAGG  GATCCCTGCAGGGCGTGCCGGTTCTGATTAAG  AGCTACGAATTCGTGCTGTCGCGAAAGTAG  CTCCTTCAATATCAAAGCTGACTGCACCAGCCATAG  AATAGAGATCTGCTAGCCTGTGCGGTGGTCTCTTTG  AAGCTTAGCTTAAGGAGGCGTTGACGATAG  ATGGCTGGTGCAGTCAGCTTTGATATTGAAGGAGC  AAGAGACCACCGCACAGGCTAGCAGATCTCTATTC  GATCTCCTGCAGGCATCCGAATCGTCCCAATCC  GATCTGGCGCGCCGCTAGATGGCGCCCTATCAC  GATCGGATCCAGATTCGCCCTTCGTATCGG  GCTAGATGGCGCCCTATCAC  GATCGAATTCTCAGACCGGCGACTGCAACC  GATCGGATCCACACGCGACCTCAGAAGAAC  GATCGAATTCACTGCTACCATATGAGTCGC  CAGTCGCCGGTCTGACTATGGCTGGTGCAGTCACG  CTGCACCAGCCATAGTCAGACCGGCGACTGCAACC  GCTGGTGCAGTCACGACACGCGACCTCAGAAGAAC  TCTGAGGTCGCGTGTCGTGACTGCACCAGCCATAG  GATCGTCGACTAGCTTCTGTGACAAGGTGG  TTTACCTGCAGGTCGAAGCCCGAAGCTGAATG  CAGTCGCCGGTCTGAATGCGAGAGGAGGGAGTATG  TCCCTCCTCTCGCATTCAGACCGGCGACTGCAACC  GATCATGCTGGGCTCACACGCGACCTCAGAAGAAC  TCTGAGGTCGCGTGTGAGCCCAGCATGATCTGAAC  GATCCCTGCAGGCTGCTGACGCATCAAAGTCC  GATCGAATTCCAAACTGCTTACCCAGACC  ATTGGAGTGCGTTGTTAGCGGCGAATGCTCTGC  GAGCATTCGCCGCTAACAACGCACTCCAATCCAG  AGTCGCCGGTCTGAAGATACCCAGAATACGTTGCC  CGTATTCTGGGTATCTTCAGACCGGCGACTGCAAC  GCGAGACGCTATACGACGCGACCTCAGAAGAACTC  CTTCTGAGGTCGCGTCGTATAGCGTCTCGCATCC  GATCGAATTCCGCCATACCGACTCCTACC  CAGACGTATTCGCCAGGTGTATGTCCAGAGGCATACGATA  GGTTGCAGTCGCCGGTCTGAGATCGTCTAATGTTGTTGTACCGTCG  TACAACAACATTAGACGATCTCAGACCGGCGACTGCAACC  TCACGTAGGCGACCATCTGAACACGCGACCTCAGAAGAAC  ATTGGCCGCAGCGGCCATTTCGAATCCTCTTGCCCAGACG  TATCGTATGCCTCTGGACATACACCTGGCGAATACGTCTG  GTTCTTCTGAGGTCGCGTGTTCAGATGGTCGCCTACGTGAC  CCGGCGCGCCCAATTGATTTCGCTGTAGCCAGATACTGTG  GCACCTGGCGCGTTGGGAAGATGGTGGGGGAGAGGAG  GGTTGCAGTCGCCGGTCTGATCATGCTGGGCTCGAACGG  GTTCTTCTGAGGTCGCGTGTTCTGAACGATTCAATTCATGACCC  CCGGCGCGCCCAATTGATTTCTGCTGACGCATCAAAGTCC  CCCCTCCTCTCCCCCACCATCTTCCCAACGCGCCAGG  ATTGGCCGCAGCGGCCATTTACTGCGCACAGCCAAGATGC  TCCGTTCGAGCCCAGCATGATCAGACCGGCGACTGCAACC  CATGAATTGAATCGTTCAGAACACGCGACCTCAGAAGAAC  TCTCGTATGCCTCGGGACATACACCTGGCGAATACGTCTG  CAGACGTATTCGCCAGGTGTATGTCCCGAGGCATACGAG  GGTTGCAGTCGCCGGTCTGACTACGGCGTCTCCAGGC  TTCGCCTGGAGACGCCGTAGTCAGACCGGCGACTGCAACC  ATTGGCCGCAGCGGCCATTTCCCAAACTGCTTACCCAGACC  AGATCTGGATTGGAGTGCATTATTAGCGGCGAATGCTCTGC  CAGAGCATTCGCCGCTAATAATGCACTCCAATCCAGATCTG  GGTTGCAGTCGCCGGTCTGATCAGTGAAGCTCATGATATGGC  CATATCATGAGCTTCACTGATCAGACCGGCGACTGCAACC  AGACGCTATACGGATACCCAACACGCGACCTCAGAAGAAC  GTTCTTCTGAGGTCGCGTGTTGGGTATCCGTATAGCGTCTC  CCGGCGCGCCCAATTGATTTTACCGACTCCTACCCTTCCTC  CTCACGTCCGTCCTCGCCATCGCCGCGCACTTCCAAATAC  GTATTTGGAAGTGCGCGGCGATGGCGAGGACGGACGTGAG  GGTTGCAGTCGCCGGTCTGACTACAGCTGGGAGGTTGAATG  ATTCAACCTCCCAGCTGTAGTCAGACCGGCGACTGCAACC  CTGACTACTAGTGGTACCCGGGGATCTTTC  CTGGATTGGAGTGCATGAATATACTGAAGATGGG  ATCTTCAGTATATTCATGCACTCCAATCCAGATC  GGCCGTGGCGATGCTCAGCTGGGAGGTTGAATGTG  AGCATCGCCACGGCCATGGTGAGCAAGGGCGAGGA  TACTAGACTAGTACCTGATATTGAAGGAGC  TGGAAAGCGGGCAGTGAG  CCATCTTCAGTATATTCGATATGCAGAACATCTTCCTCGC  ATGGCCGTGGCGATGCTGATGGCAGACGCAGTGATGTTGTG  CCATCTTCAGTATATTCGATATGGCGAGGACGGACGTG  ATGGCCGTGGCGATGCTGATCAGCTGGGAGGTTGAATGTG  GACGACCGACTCCTTTGATG  GGCAATGGGCTCTTCTCTGG  ATCGTTCTCGCGAAGGCAGC  CGACCACAATGACGCCATTC  GACGCCATTCTCGACAGTCC  CTATGCAAGCGCTGGCTCCG  CTGAGTGATGCCCATGAC  CATGATATGGCATCAATGG  TCCCTGGACCCGAGAATG  CGCGACCATTTGATCGTC  ACCTGTGAAGTTGCCTAC  GTTGTTGTACCGTCAGCC  GTGAAGTTTCCCACAGGC | | | | *Zt80707* deletion – Hyg-R [F]  *Zt80707* deletion – Hyg-R [R]  *Zt80707* deletion – UF [F]  *Zt80707* deletion – UF [R]  *Zt80707* deletion – DF [F]  *Zt80707* deletion – DF [R]  *Zt103264* qRT-PCR [F]  *Zt103264* qRT-PCR [R]  *Zt80707* qRT-PCR [F]  *Zt80707* qRT-PCR [R]  *Zt99044*(GAPDH) qRT-PCR [F]  *Zt99044*(GAPDH) qRT-PCR [R]  *Zt89160* qRT-PCR [F]  *Zt89160* qRT-PCR [R]  *Zt110804* qRT-PCR [F]  *Zt110804* qRT-PCR [R]  *Zt89160* deletion – UF [F]  *Zt89160* deletion – UF [R]  *Zt89160* deletion – Hyg-R [F]  *Zt89160* deletion – Hyg-R [R]  *Zt89160* deletion – DF [F]  *Zt89160* deletion – DF [R]  *Zt110804* deletion – UF [F]  *Zt110804* deletion – UF [R]  *Zt110804* deletion – Hyg-R [F]  *Zt110804* deletion – Hyg-R [R]  *Zt110804* deletion – DF [F]  *Zt110804* deletion – DF [R]  *Zt103264* deletion – UF [F]  *Zt103264* deletion – UF [R]  *Zt103264* deletion – DF [F]  *Zt103264* deletion – DF [R]  *Zt103264* deletion – Hyg-R [F]  *Zt103264* deletion – Hyg-R [R]  *Zt80707* complementation – UF [F]  *Zt80707* complementation – UF [R]  *Zt80707* complementation – DF [F]  *Zt80707* complementation – DF [R]  *Zt80707* complementation – G418-R [F]  *Zt80707* complementation – G418-R [R]  *Zt103264* complementation – UF [F]  *Zt103264* complementation – UF [R]  *Zt103264* complementation – G418-R [F]  *Zt103264* complementation – G418-R [R]  *Zt103264* complementation – DF [F]  *Zt103264* complementation – DF [R]  *Zt89160* complementation – UF [F]  *Zt89160* complementation – UF [R]  *Zt89160* complementation – G418-R [F]  *Zt89160* complementation – G418-R [R]  *Zt89160* complementation – DF [F]  *Zt89160* complementation – DF [R]  *Zp80707* replacement – UF [F]  *Zp80707* replacement – UF [R]  *Zp80707* replacement – ORF [F]  *Zp80707* replacement – ORF [R]  *Zp80707* replacement – G418-R [F]  *Zp80707* replacement – G418-R [R]  *Zp80707* replacement – DF [F]  *Zp80707* replacement – DF [R]  *Zp13_103264* replacement – ORF [F]  *Zp13_103264* replacement – ORF [R]  *Zp13_103264* replacement – G418-R [F]  *Zp13_103264* replacement – G418-R [R]  *Zp13_103264* replacement – UF [F]  *Zp13_103264* replacement – UF [R]  *Zp13_103264* replacement – DF [F]  *Zp13_103264* replacement – DF [R]  *Zp89160* replacement – ORF [F]  *Zp89160* replacement – ORF [R]  *Zp89160* replacement – DF [F]  *Zp89160* replacement – DF [R]  *Zp89160* replacement – UF [F]  *Zp89160* replacement – UF [R]  *Zp89160* replacement – G418-R [F]  *Zp89160* replacement – G418-R [R]  *Zp14_103264* replacement – UF [R]  *Zp14_103264* replacement – ORF [F]  *Zp14_103264* replacement – ORF [R]  *Zp14_103264* replacement – G418-R [F]  *Za80707* replacement – UF [F]  *Za80707* replacement – UF [R]  *Za80707* replacement – ORF [F]  *Za80707* replacement – ORF [R]  *Za80707* replacement – G418-R [F]  *Za80707* replacement – G418-R [R]  *Za80707* replacement – DF [F]  *Za80707* replacement – DF [R]  *Zp80707*-SP replacement – UF [R]  *Zp80707*-SP replacement – ORF [F]  *Zp80707*-SP replacement – ORF [R]  *Zp80707*-SP replacement – G418-R [F]  Secretion assay *Zt80707 -* pgpdA [F]  Secretion assay *Zt80707 -* pgpdA [R]  Secretion assay *Zt80707 -* ORF [F]  Secretion assay *Zt80707 -* ORF [R]  Secretion assay *Zt80707 -* backbone [F]  Secretion assay *Zt80707 -* backbone [R]  Secretion assay *Zt77228 -* backbone [F]  Secretion assay *Zt111221* (LysM) [F]  Secretion assay *Zt111221* (LysM) [R]  Secretion assay *Zp80707* [F]  Secretion assay *Zp80707* [R]  5’RACE-PCR *Zt80707* – synthesis [R]  5’RACE-PCR *Zt80707* – GSP [R]  5’RACE-PCR *Zt80707* – NGSP [R]  5’RACE-PCR *Zt103264* – synthesis [R]  5’RACE-PCR *Zt103264* – GSP [R]  5’RACE-PCR *Zt103264* – NGSP [R]  5’RACE-PCR *Zp80707* – GSP [R]  5’RACE-PCR *Za80707* – synthesis [R]  5’RACE-PCR *Za80707* – GSP [R]  5’RACE-PCR *Zp103264* – synthesis [R]  5’RACE-PCR *Zp103264* – NGSP [R]  5’RACE-PCR *Za103264* – synthesis [R]  5’RACE-PCR *Za103264* – NGSP [R] |

^1^ Sequences are shown in 5‘ to 3‘ direction

^2^ Oligonucleotides hybridize with the sense-strand [R] or with the complementary strand [F] of the corresponding gene.
