# Supplementary material for: Rapidly Evolving Genes Are Key Players in Host Specialization and Virulence of the Fungal Wheat Pathogen Zymoseptoria tritici (Mycosphaerella graminicola)
Source: PLoS Pathog. 2015 Jul 30;11(7):e1005055. doi: 10.1371/journal.ppat.1005055 (PMC4520584; doi:10.1371/journal.ppat.1005055)
Supplement: S2 Table — (DOCX) [file ppat.1005055.s002.docx]

**Supplementary Table 3**

| Plasmids used in this study | |  |  |  | |
| --- | --- | --- | --- | --- | --- |
| Name | Application | | | | Reference |
| pES23  pES46  pES64  pES65  pES74  pES86  pES87  pES98  pES99  pES126  pES127  pES143  pES150 | *Zt80707* deletion (Hyg-R)  *Zt80707* complementation (G418-R)  *Zt89160* deletion (Hyg-R)  *Zt110804* deletion (Hyg-R)  *Zt103264* deletion (Hyg-R)  *Zt103264* complementation (G418-R)  *Zt89160* complementation (G418-R)  *Zp80707* (Zp13) replacement (G418-R)  *Za80707* (Za17) replacement (G418-R)  *Zp89160* (Zp13) replacement (G418-R)  *Zp103264* (Za17) replacement (G418-R)  *Zp80707*-SP (Zp13) replacement (G418-R)  Secretion assay (G418-R) | | | | This study  This study  This study  This study  This study  This study  This study  This study  This study  This study  This study  This study  This study |
