# Supplementary figures and images for: Rapidly Evolving Genes Are Key Players in Host Specialization and Virulence of the Fungal Wheat Pathogen Zymoseptoria tritici (Mycosphaerella graminicola)
Source: PLoS Pathog. 2015 Jul 30;11(7):e1005055. doi: 10.1371/journal.ppat.1005055 (PMC4520584; doi:10.1371/journal.ppat.1005055)

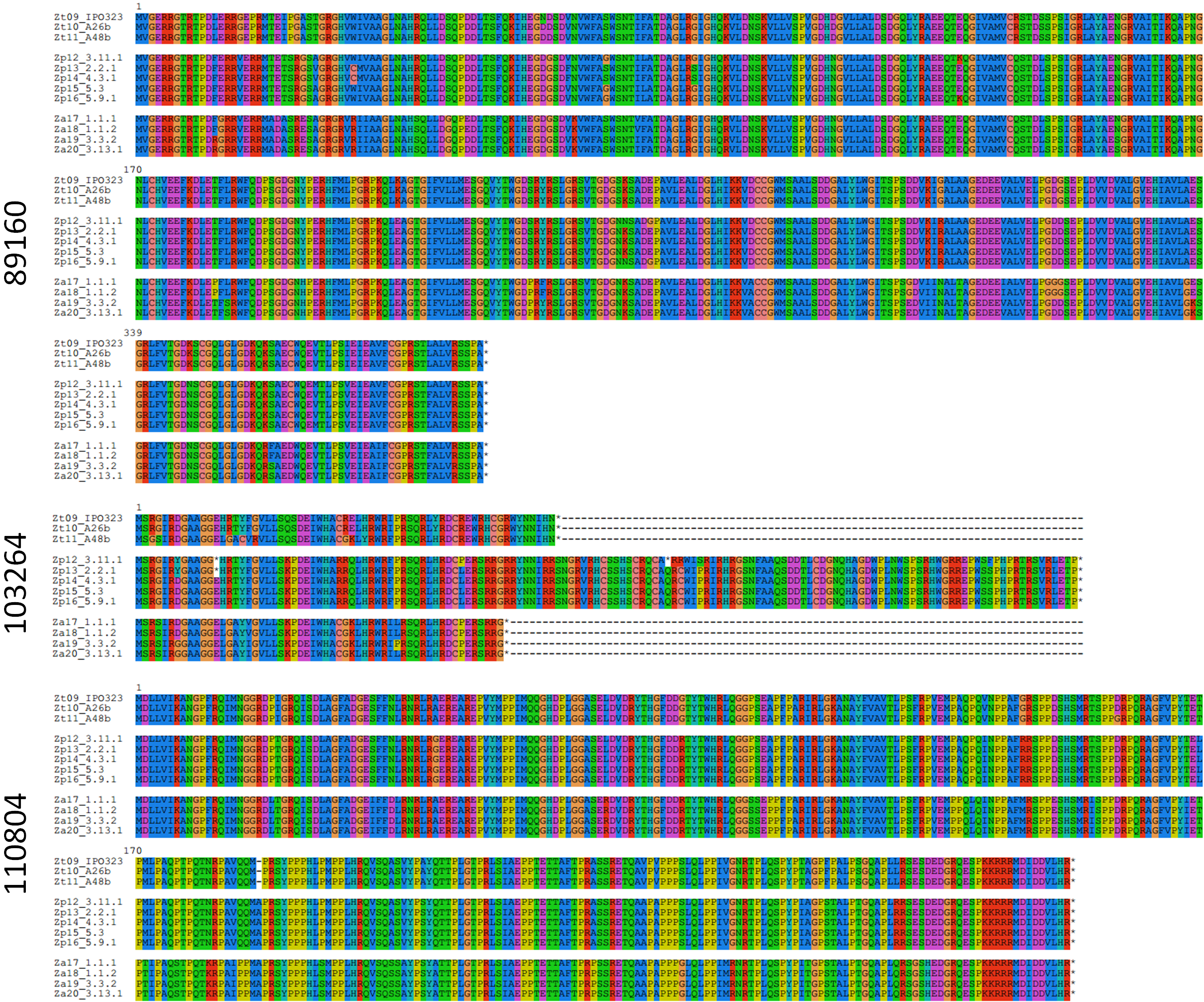

Supplement: S1 Fig — Protein alignment of Zt89160, Zt103264 and Zt110804 of different isolates of Z. tritici (Zt), Z. pseudotritici (Zp) and Z. ardabiliae (Za). (TIF) [file ppat.1005055.s003.tif]

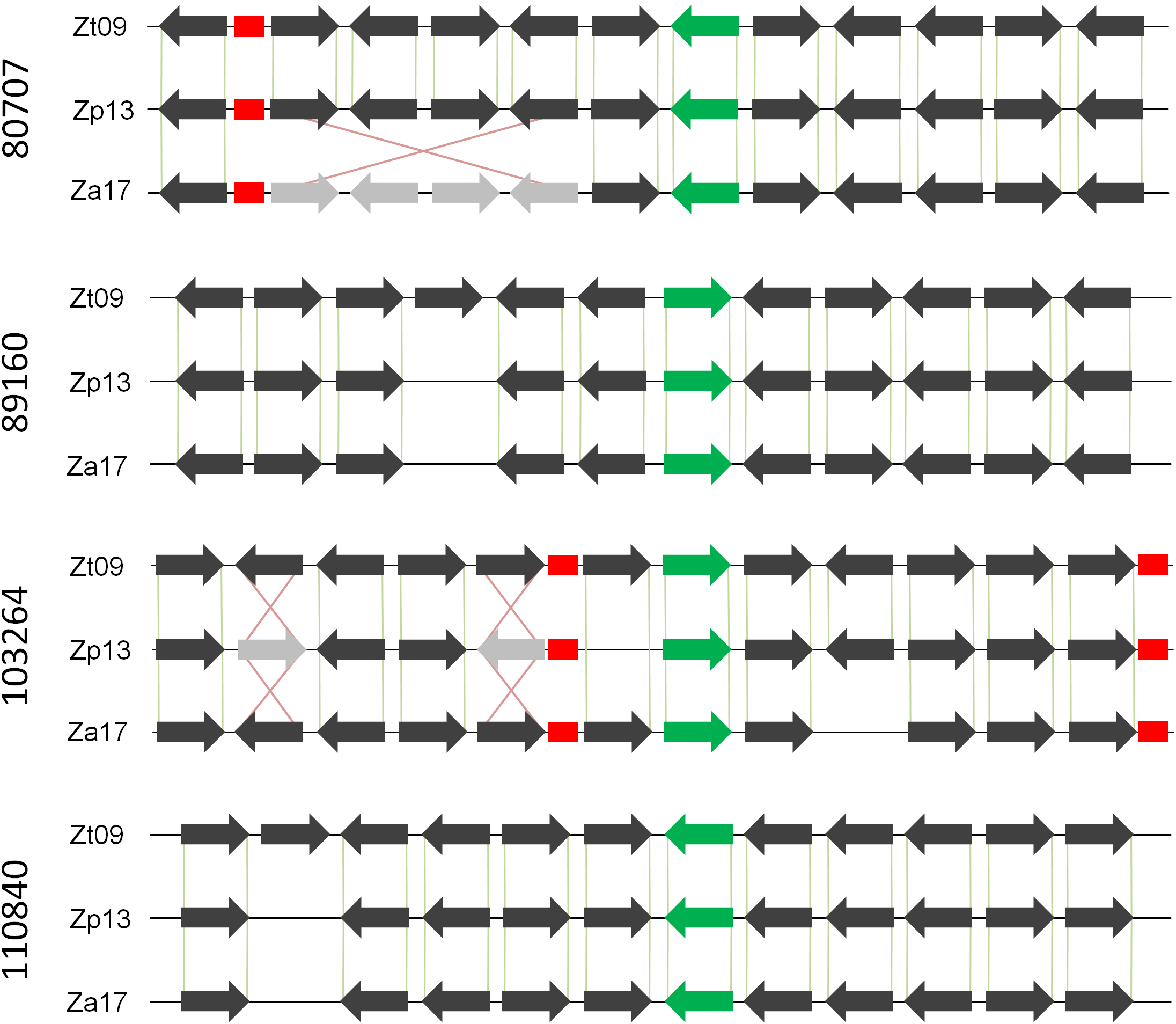

Supplement: S2 Fig — Schematic illustration of conserved gene order in genomic regions encoding Zt80707, Zt89160, Zt103264 and Zt110804 in Z. tritici (Zt09), Z. pseudotritici (Zp13) and Z. ardabiliae (Za17). The four candidate genes are shown in green, neighboring genes are shown in dark grey, inverted genes are shown in light grey and transposable elements are shown in red. (TIF) [file ppat.1005055.s004.tif]

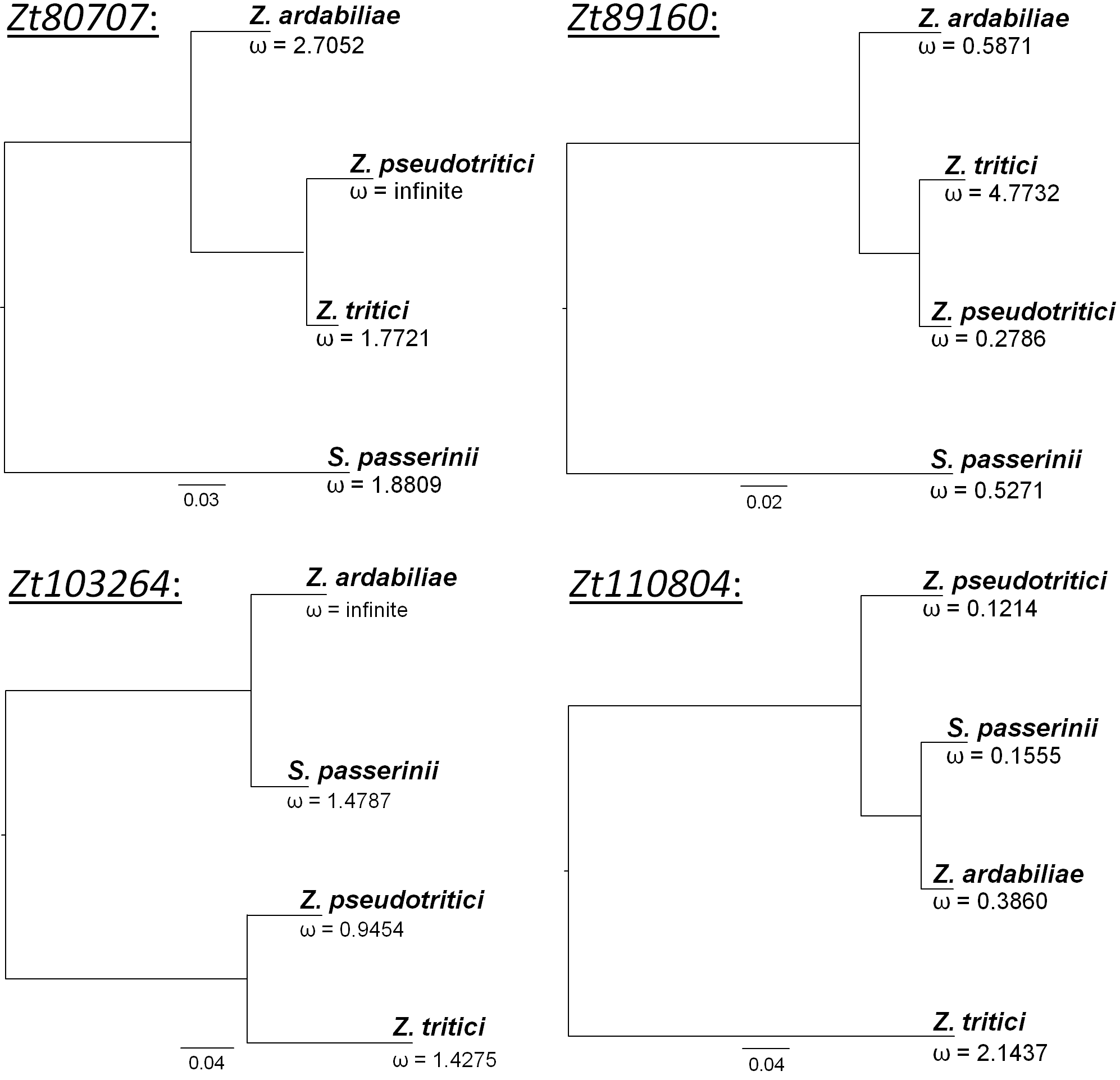

Supplement: S3 Fig — Phylogenetic trees were made for Z. tritici genes Zt80707 (A), Zt89160 (B), Zt103264 (C) and Zt110804 (D) and the respective orthologs of Z. pseudotritici, Z. ardabiliae and Z. passerinii. A branch model was used to estimate the ω (d N/d S ratio) for each branch. Branch-specific ω values above 1 are indicative of positive selection. (TIF) [file ppat.1005055.s005.tif]

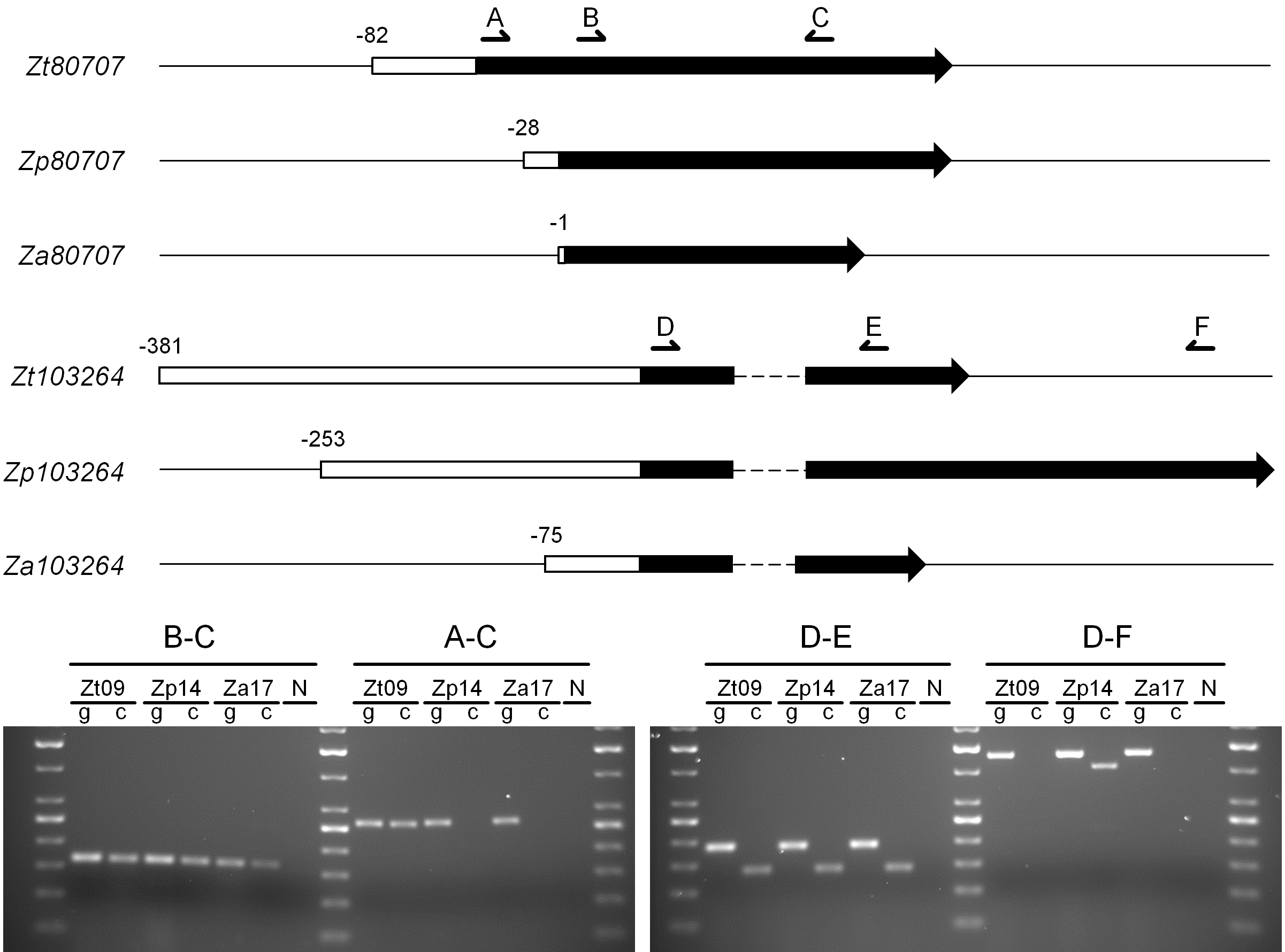

Supplement: S4 Fig — Rapid Amplification of cDNA Ends (RACE-PCR) of Zt80707 and Zt103264 revealed the ORFs (black arrows) schematically illustrated, including 5’UTR (white rectangles) and start codon positions. The lengths of the ORFs were confirmed using the primer combinations “A” and “C” and “B” and “C” with cDNA of Zt80707 and D-E and D-F of Zt103264. As template for the PCRs genomic DNA (g) of the three strains, Zt09, Zp14 and Za17 were used, as well as cDNA (c) obtained from infected wheat leaves. (TIF) [file ppat.1005055.s006.tif]

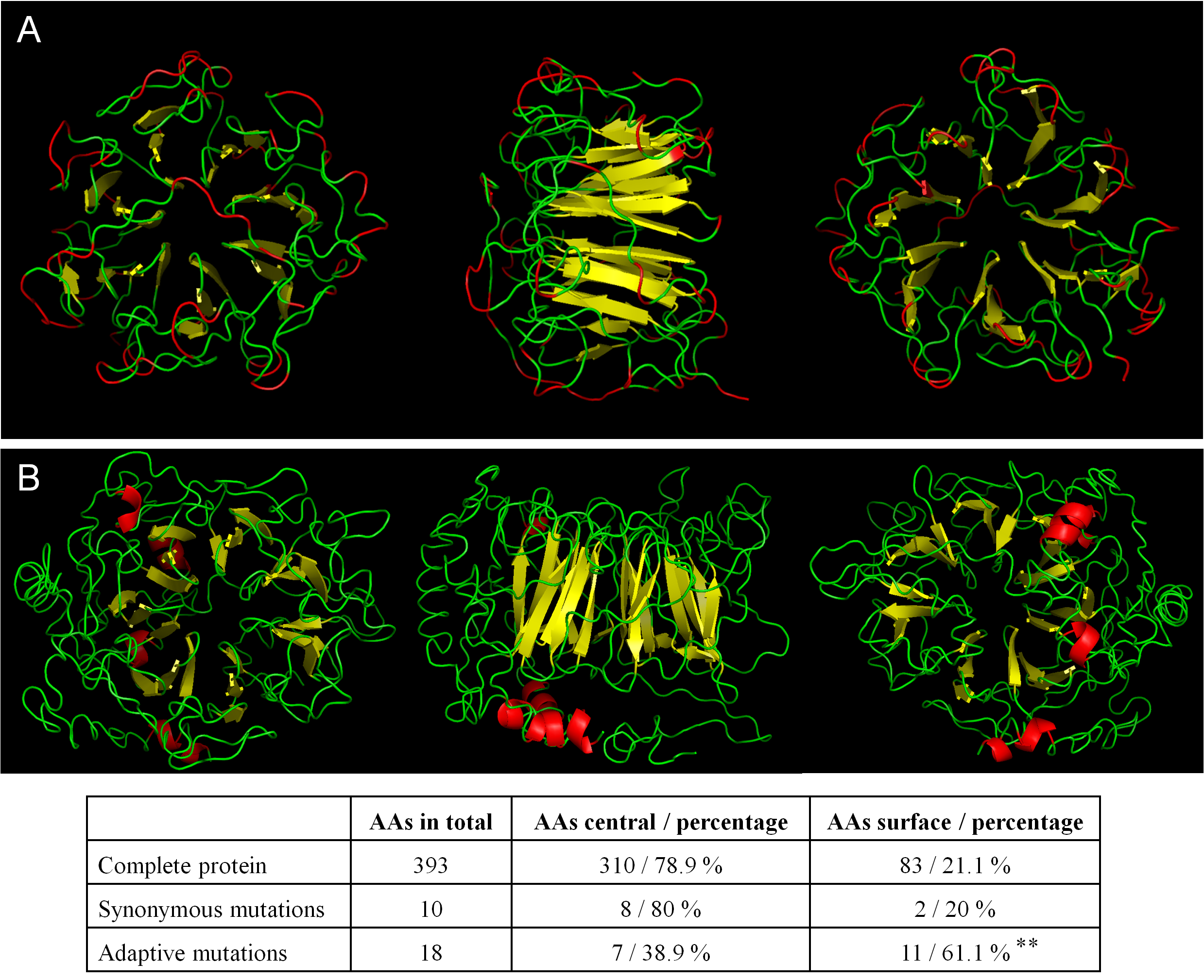

Supplement: S5 Fig — A) Top view, side view and bottom view of the Zt89160 protein structure predicted with I-TASSER. Beta sheets are depicted in yellow and the accessible amino acids on the surface of the protein are shown in red. B) Top view, side view and bottom view of the structure predicted by I-TASSER for the Drosophila melanogaster protein CG6678, a protein homologous to Zt89160. Beta sheets are depicted in yellow and alpha helices are shown in red. The table shows that the significant majority of positively selected amino acids is located on the surface of the protein Zt89160. ** p<0.01. p-value calculated using a Fishers exact test. (TIF) [file ppat.1005055.s007.tif]

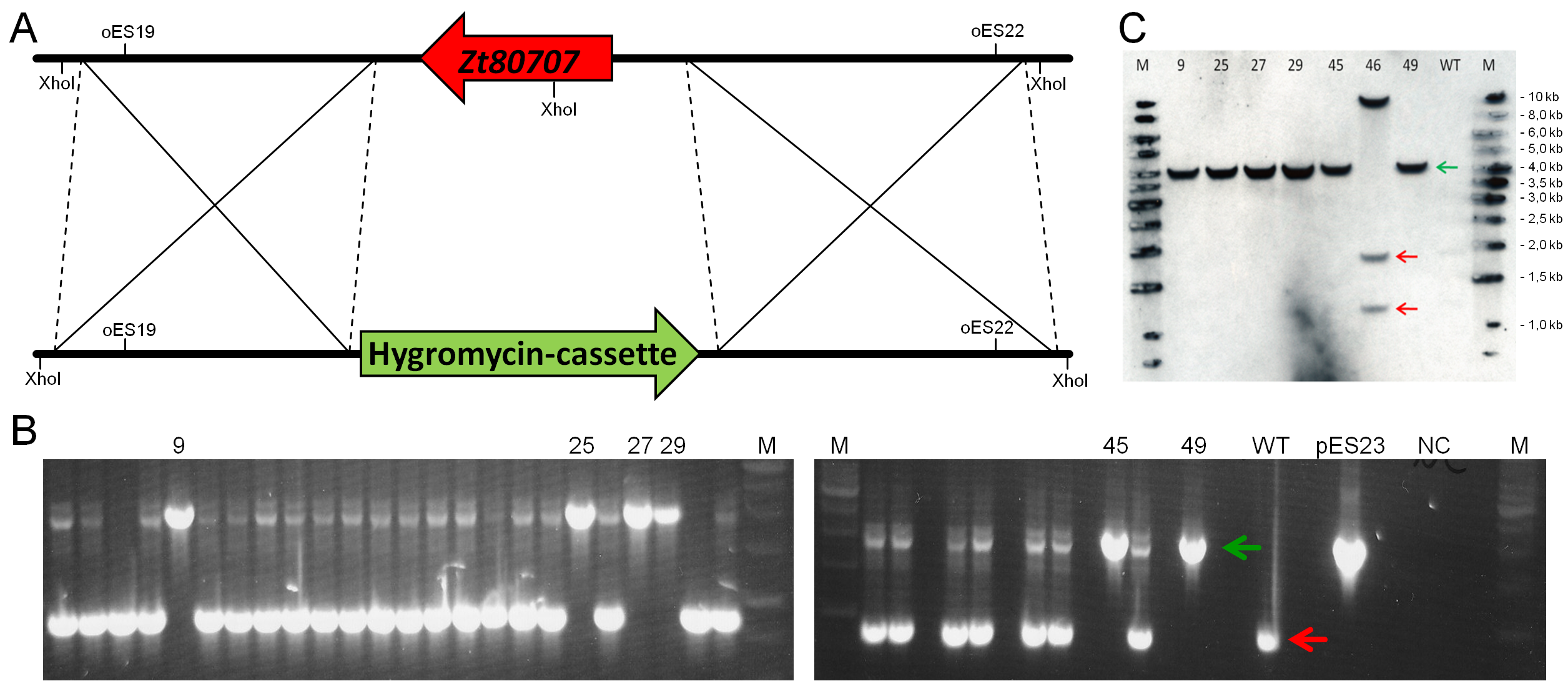

Supplement: S6 Fig — Schematic illustration of the gene deletion approach in Z. tritici (example Zt80707). A) Gene deletion by homologous recombination using Agrobacterium tumefaciens-mediated transformation. B) PCR screening to identify correctly transformed strains using the primer combination oES19 and oES22. C) Correctly transformed strains were finally confirmed via Southern blot analysis using the oES19- and oES22-amplified sequence as a probe for hybridization. (TIF) [file ppat.1005055.s008.tif]

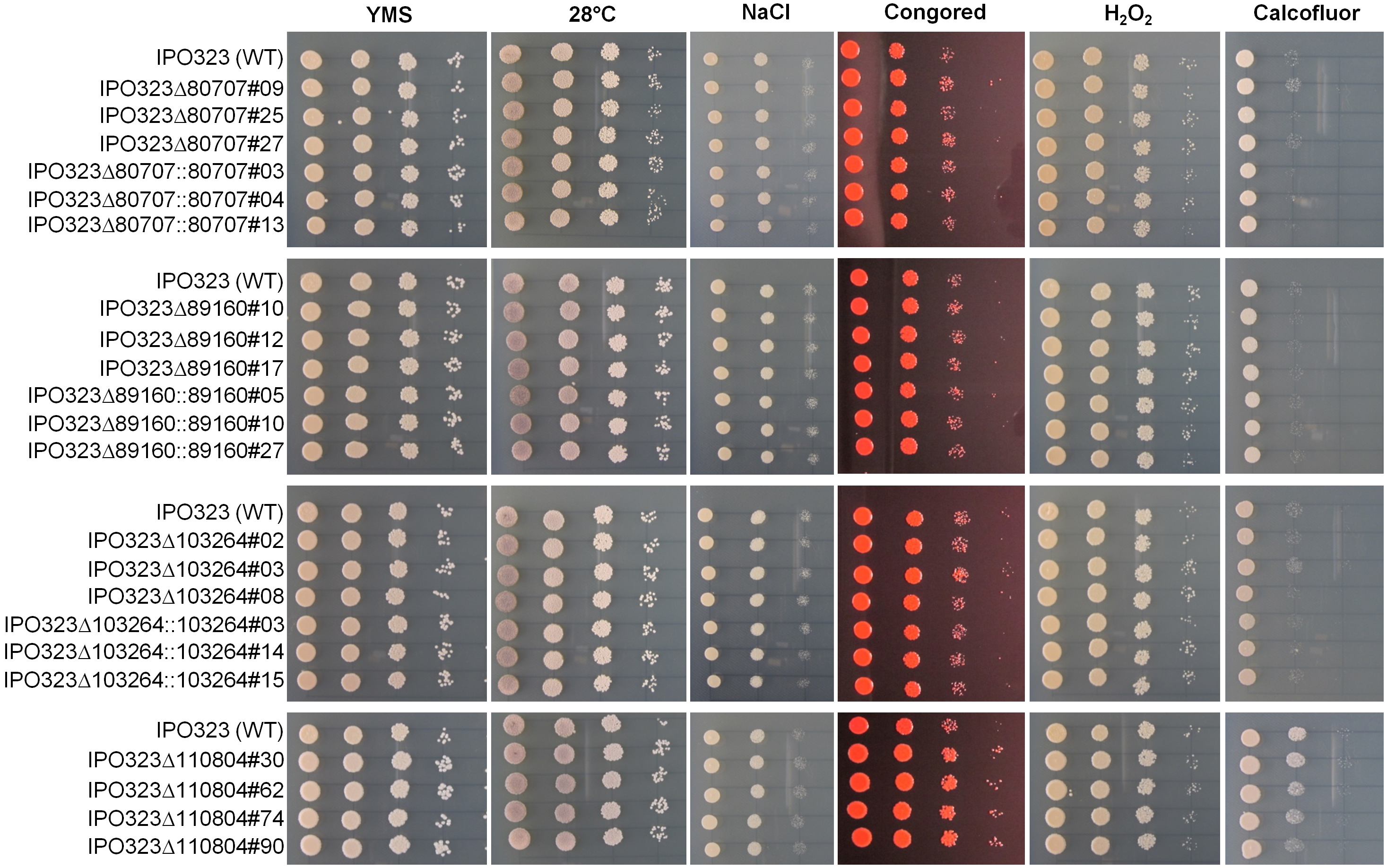

Supplement: S7 Fig — Three independent mutants of each generated deletion and complementation strain for the genes Zt80707, Zt89160 and Zt103264 were tested under multiple abiotic stress conditions. The deletion of Zt110804 was not complemented. The following conditions were used: heat stress (28°C), NaCl (1.5 M), Congored (500 μg/ml), H2O2 (2 mM) and Calcofluor (200 μg/ml). (TIF) [file ppat.1005055.s009.tif]

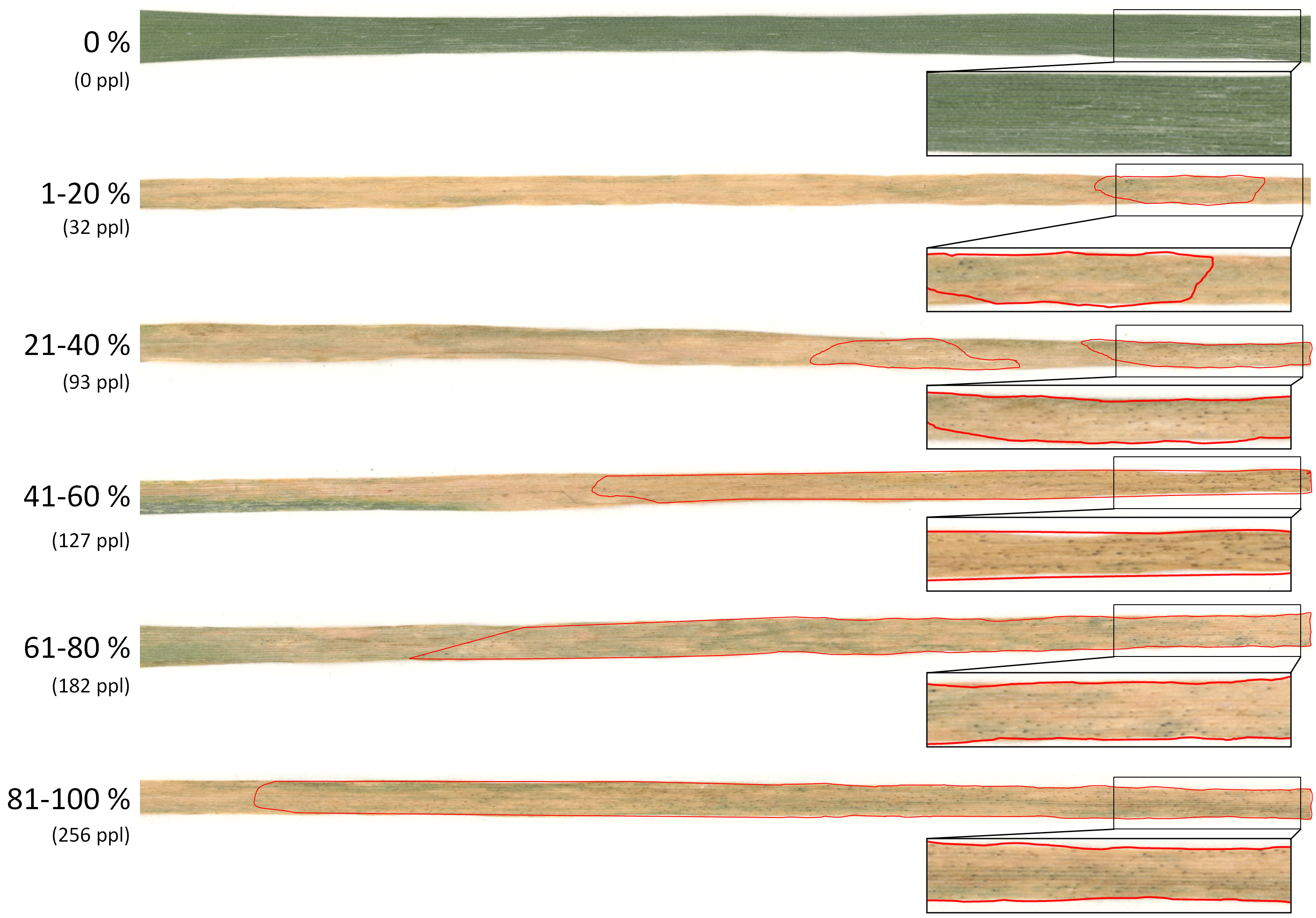

Supplement: S8 Fig — Representative images for the six categories that were used to score disease levels at 28 dpi in plant assays. The number of pycnidia per leaf (ppl) is shown for every leaf. Areas encircled by red lines illustrate the proportion of necrotic areas with pycnidia. (TIF) [file ppat.1005055.s010.tif]

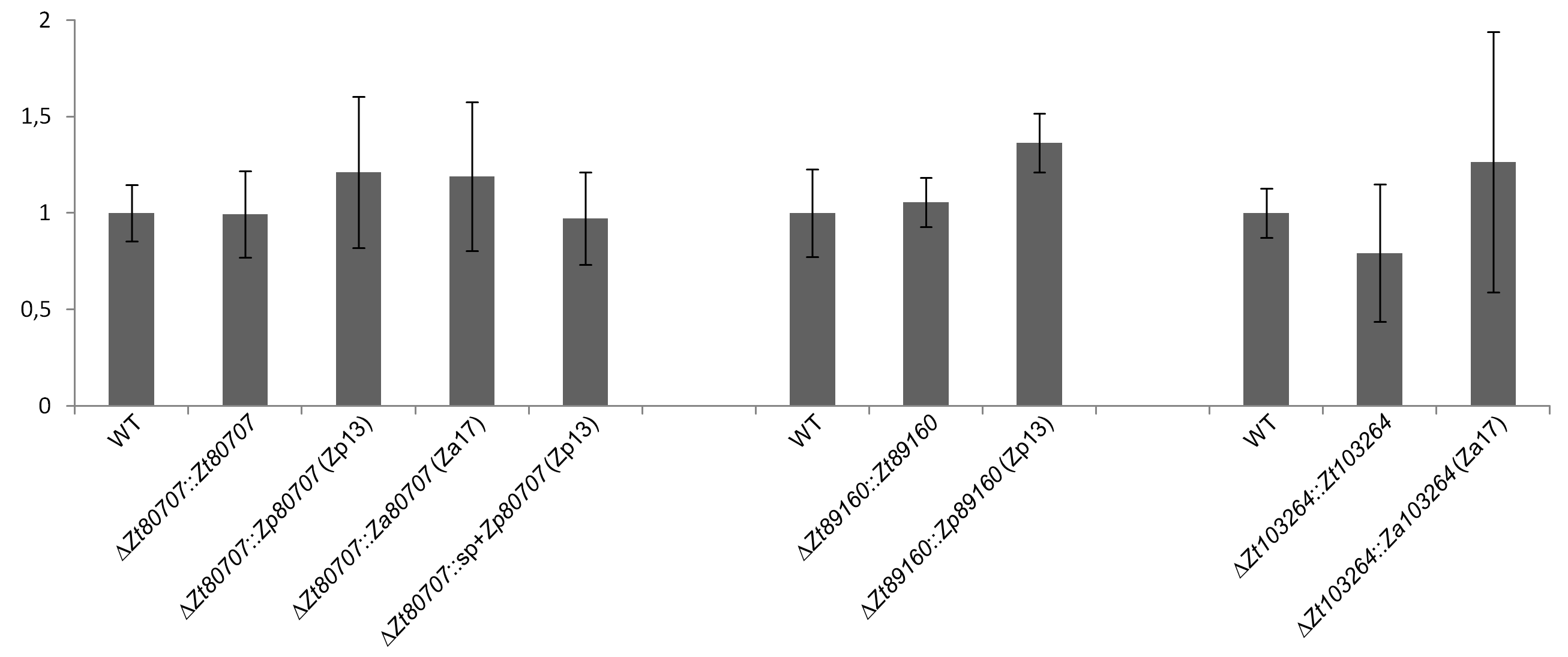

Supplement: S9 Fig — Expression levels of the three genes in the respective complementation- and replacement strains at 4 dpi have been compared to wild-type Z. tritici infected leaves (WT). Values are normalized to the expression of the gene encoding GAPDH, a constitutively expressed housekeeping control. Error bars indicate the standard error of the mean (SEM) of three independent biological replicates per sample. (TIF) [file ppat.1005055.s011.tif]

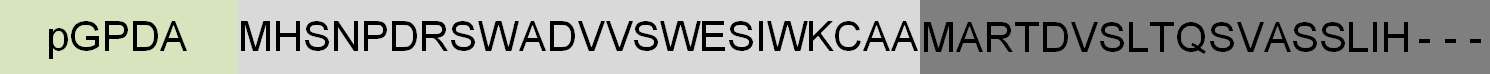

Supplement: S10 Fig — The promoter (light green) and signal peptide (light grey) of Zt80707 from Z. tritici were fused with the ORF of Zp80707 from Z. pseudotritici (beginning of the ORF shown in dark grey) to obtain the secretion of Zp80707 in Z. tritici. (TIF) [file ppat.1005055.s012.tif]

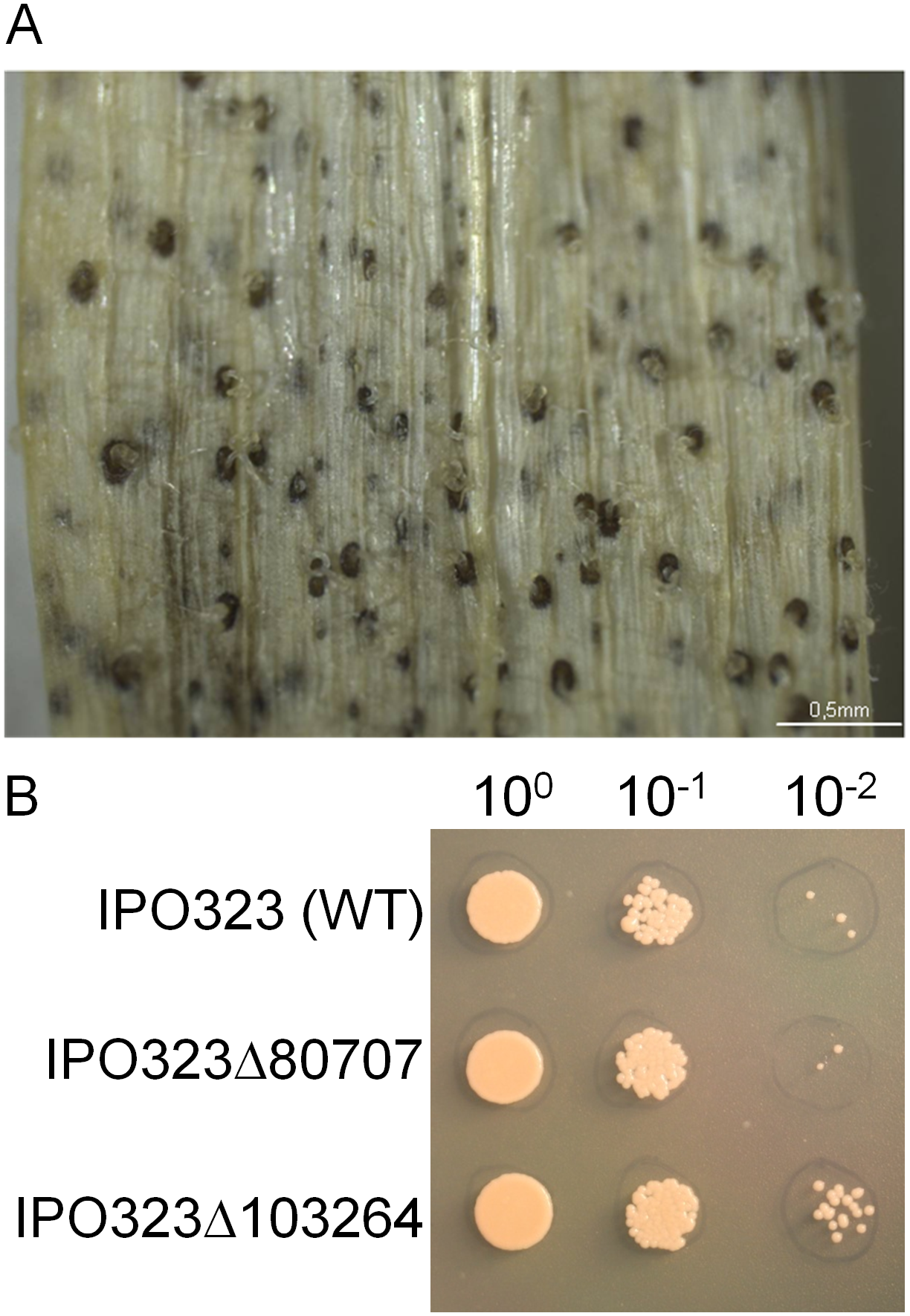

Supplement: S11 Fig — A) Macroscopic picture of oozing pycnidia of an infected wheat leaf (IPO323 wild-type) after surface sterilization and one week of incubation in high humidity. B) Exuded fungal spores were isolated and added in different concentrations to YMS media to test the viability of wild-type and mutant spores. (TIF) [file ppat.1005055.s013.tif]
